# Theoretical explanations for maintenance of behaviour change: a systematic review of behaviour theories

**DOI:** 10.1080/17437199.2016.1151372

**Published:** 2016-03-07

**Authors:** Dominika Kwasnicka, Stephan U Dombrowski, Martin White, Falko Sniehotta

**Affiliations:** ^a^ Institute for Health and Society, Newcastle University, Newcastle upon Tyne, UK; ^b^ UKCRC Centre for Excellence in Translational Public Health Research (Fuse), Newcastle University, Newcastle upon Tyne, UK; ^c^ School of Natural Sciences, University of Stirling, Stirling, UK; ^d^ UKCRC Centre for Diet and Activity Research (CEDAR), University of Cambridge, Cambridge, UK

**Keywords:** Behaviour change, behaviour maintenance, theory, theory review

## Abstract

***Background:*** Behaviour change interventions are effective in supporting individuals in achieving temporary behaviour change. Behaviour change maintenance, however, is rarely attained. The aim of this review was to identify and synthesise current theoretical explanations for behaviour change maintenance to inform future research and practice.

***Methods:*** Potentially relevant theories were identified through systematic searches of electronic databases (Ovid MEDLINE, Embase, PsycINFO). In addition, an existing database of 80 theories was searched, and 25 theory experts were consulted. Theories were included if they formulated hypotheses about behaviour change maintenance. Included theories were synthesised thematically to ascertain overarching explanations for behaviour change maintenance. Initial theoretical themes were cross-validated.

***Findings:*** One hundred and seventeen behaviour theories were identified, of which 100 met the inclusion criteria. Five overarching, interconnected themes representing theoretical explanations for behaviour change maintenance emerged. Theoretical explanations of behaviour change maintenance focus on the differential nature and role of motives, self-regulation, resources (psychological and physical), habits, and environmental and social influences from initiation to maintenance.

***Discussion:*** There are distinct patterns of theoretical explanations for behaviour change and for behaviour change maintenance. The findings from this review can guide the development and evaluation of interventions promoting maintenance of health behaviours and help in the development of an integrated theory of behaviour change maintenance.

## Introduction

### Importance of behaviour maintenance

There is considerable evidence that behaviour can be effectively modified through behaviour change interventions (Albarracin et al., 2005; Hobbs et al., 2013). However, evidence for the sustainability of behaviour change in response to interventions is limited (Avenell et al., 2004; Carpenter et al., 2013; Dombrowski, Knittle, Avenell, Araújo-Soares, & Sniehotta, 2014; Fjeldsoe, Neuhaus, Winkler, & Eakin, 2011). This is partly because few studies evaluate long-term effects and partly because intervention effects diminish over time (Curioni & Lourenco, 2005; Dombrowski, Avenell, & Sniehotta, 2010). Relapse rates are high for individuals who join weight loss programmes (Tsai & Wadden, 2005); initiate smoking cessation attempts (Carpenter et al., 2013; Hughes, Keely, & Naud, 2004); try to reduce alcohol consumption (Moos & Moos, 2006) or make attempts to stop sexual risk behaviours (Kelly, Stlawrence, & Brasfield, 1991). Theory of behaviour change maintenance can provide guidance on the development and evaluation of interventions promoting sustained change in health behaviours. Current evidence about the effectiveness of theory-based interventions to change health-related behaviours is inconsistent (Gourlan et al., 2015; Prestwich et al., 2014) which may, in part, be due to the lack of theoretical elaboration on the process of maintenance after initial change.

### Conceptual background to the analysis of behaviour change maintenance theories

**Figure 1.**
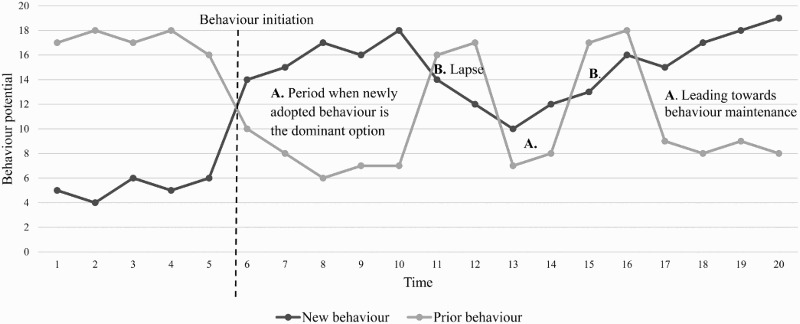
Changes in behaviour potential (likelihood of engaging in a particular behaviour) over time following initial behaviour change.

To facilitate the review and synthesis of a wide range of theories with varied objectives, it is helpful first to briefly rehearse their conceptual background in the psychology of behaviour. In any given situation individuals have various behavioural options. These behavioural options might be intentionally and/or impulsively driven and may be predicated by prior behaviour. Each of these options has a certain likelihood of being enacted at any given time, reflecting current individual (motivation, habits, resources) and contextual (cues, opportunity costs and opportunities) factors. This likelihood of enacting each behavioural response at a given time and in a given context has been termed a ‘behavioural potential’ by Rotter (1960). Similarly, Heckhausen and Beckmann (1990) conceptualised the variable feature of how strongly an intention is predisposed for implementation in a given situation as ‘fiat tendencies’. Behavioural potentials vary over time and context as illustrated in a fictional example in Figure 1. The individual is hypothesised to enact the behavioural option with the highest potential in each situation and time (Heckhausen & Beckmann, 1990; Rotter, 1960).

Before behaviour change, prior behaviour, such as unhealthy eating patterns, may be the dominant response across times and contexts. After behaviour change, newly adopted behaviour may become the dominant response in many contexts. Area A in Figure 1 illustrates times when enacting the newly adopted behaviour is the dominant response, area B shows times when the prior behaviour becomes dominant again, indicating the risk of a potential lapse. The new behaviour is likely to be maintained over time if it becomes the dominant response across contexts. Thus, a theoretical analysis of behaviour change maintenance will need to take into consideration various behavioural options and the probability of them being enacted over time and across contexts. It is currently unclear what conditions are required to maintain the new behaviour and prevent relapse, or to re-establish the new behaviour after relapse.

### Theory and behaviour change maintenance

For the purpose of this review, theory was defined as a set of statements that organises, predicts and explains observations. Theory explains how phenomena relate to each other, and what can be expected under unknown conditions (Bem & Looren de Jong, 1997). Theory may be useful for research to improve our understanding of maintained behaviour as well as for the design and implementation of interventions to achieve behaviour change maintenance (Craig et al., 2008). To date there has been no comprehensive review or synthesis of theories to explain behaviour change maintenance. The aim of this review was therefore to identify and synthesise theoretical explanations for behaviour change maintenance from behavioural theories and to examine the relationships between these explanations. Theories for maintenance of any human behaviour were examined; however, our particular interest was in the application of the findings to health behaviours. The main research question was what are the current theoretical explanations for behaviour change maintenance?

## Methods

### Design

Systematic review of behaviour theories. Table 1 provides a summary of methods.

**Table 1.** A summary of methods.

Structured theory review – methods summary	
1. Theory identification	From three data sources:
	Systematic on-line search ‘Theory Project’ list of 80 theories Contact with 40 experts (25 replied)
2. Inclusion/exclusion	Criteria and definition of terms
3. Data extraction	Including: ID number; theory name, full reference; verbatim general statements about behaviour change maintenance (direct quote) and explanations of behaviour change maintenance (interpretation); intended theoretical application; specified population and/or behaviour
4. Narrative data synthesis	(A) Testable explanations for behaviour change maintenance reviewed in included theories (B) Generation of themes: testable explanations analysed and grouped into theory themes and subthemes (C) Theory themes validated (10 health psychology researchers)
5. Review writing	Review structured around the themes; interconnections between the themes discussed

### Theory identification

Theories were identified in three ways:

- Systematic database searches were performed to identify theories which met the inclusion criteria (see below). Ovid MEDLINE, Embase and PsycInfo databases were searched using a comprehensive search strategy (Appendix 1), a maintenance-relevant set of search terms included ‘maintenance’, ‘behaviour maintenance’, ‘maintain’, ‘sustain’, ‘sustained behaviour’, ‘sustained change’, ‘habit' and ‘maintenance stage’ as well as appropriate synonyms, and American spellings, adjusted in accordance with the particular database. There was no time limit applied. The search was updated on 1 March 2014.
- Eighty theories from a systematic review of behaviour change theories across psychology, sociology, anthropology and economics were reviewed for inclusion (Michie, Campbell, Brown, & West, 2014).^1^
- Forty behaviour change theory experts were contacted and asked to identify any theories relevant to behaviour change maintenance; experts were defined as theory authors or critics.
- For each theory, key articles were identified based on relevance and citations (Appendix 2).

### Inclusion and exclusion criteria

*Inclusion criteria:* Theories published in any language before 1 March 2014 related to behaviour change maintenance based on the following operational definitions (‘theory’ and ‘behaviour’ definitions taken from Michie et al., 2014):

*Theory*: A set of concepts and/or statements with specification of how phenomena relate to each other. Theory provides an organising description of a system that accounts for what is known, and explains and predicts phenomena.

*Behaviour*: Anything an individual does in response to internal or external events. Overt action (motor or verbal) which is directly measurable; behaviours are physical events that occur in the body and are controlled by the brain.

*Behaviour change maintenance*: The continuous performance of a behaviour following an initial intentional change at a level that significantly differs from the baseline performance in the intended direction. While some authors have suggested time cut-offs for behaviour change and transition to behaviour change maintenance (Prochaska & Di Clemente, 1983; Prochaska, DiClemente, & Norcross, 1992), a time cut-off was not indicated here, as they may vary depending on behaviour, context and individual factors.

*Exclusion criteria*: (a) Theories about animal behaviour; (b) theories exclusively based on research with animals and (c) unpublished theories presented in dissertations and doctoral theses. Screening was conducted by two reviewers. All cases where inclusion/exclusion was unclear were discussed and agreed within the research team.

### Data extraction

Each theory was allocated a unique ID (Appendix 3). The theory name and its full references were recorded. Initially, verbatim statements about behaviour change maintenance and explanations of behaviour change maintenance based on interpretations of the theoretical statements were extracted from each theory using a standardised data extraction form. In addition, intended theoretical applications including behaviour, context or population specificity were extracted if stated. Data was extracted by one reviewer and two other reviewers each independently extracted data from 10% of the included articles. Extraction forms were compared resulting in full agreement between the reviewers.

### Data synthesis

Explanatory hypotheses for behaviour change maintenance were initially extracted by one researcher. The data synthesis team met regularly over 18 months to discuss each of the theories from the dataset and confirm the extracted theory statements. The data synthesis team consisted of the authors with backgrounds in psychology and public health who have experience in researching and applying behavioural theories, and in conducting systematic reviews and qualitative synthesis. For each theory, statements about maintenance were extracted verbatim and transformed into simple, jargon-free explanatory hypotheses about maintenance. This allowed for the grouping of similar explanatory hypotheses across theories. The outcome of the meetings was a list of summative explanatory hypotheses about behaviour change maintenance derived from all included theories.

Thematic theoretical analysis of the explanatory hypotheses was employed to synthesise the data and identify patterns of theoretical explanations (themes). The analysis followed a staged process including familiarisation with quotes and their interpretations, generating initial codes (specific themes and subthemes), assessing themes among codes (overlapping characteristics), reviewing themes, defining and naming themes and producing a final report. After theory themes were generated overlaps and relationships between themes were analysed. Their relationship and dependencies are described in the discussion. Thematic analysis was used to generate a concise set of summative explanatory constructs and propositions about their role in the maintenance of behaviour change. These summative constructs represent broad themes based on commonalities between theoretical explanations in terms of constructs and assumed mechanisms. The theoretical propositions within each theme were further organised by subthemes, illustrated by subheaders in the results section.

The themes resulting from the data synthesis were cross-validated by 10 health psychology researchers not familiar with the current review. They evaluated the validity of themes by allocating each of a randomly selected 10% of the total extracted theoretical statements to themes (Appendix 4). Allocation of one statement to more than one theme was permitted. Inter-rater agreement on allocation of themes was calculated as Krippendorff's *α* (Krippendorff, 2012).

## Results

**Figure 2.**
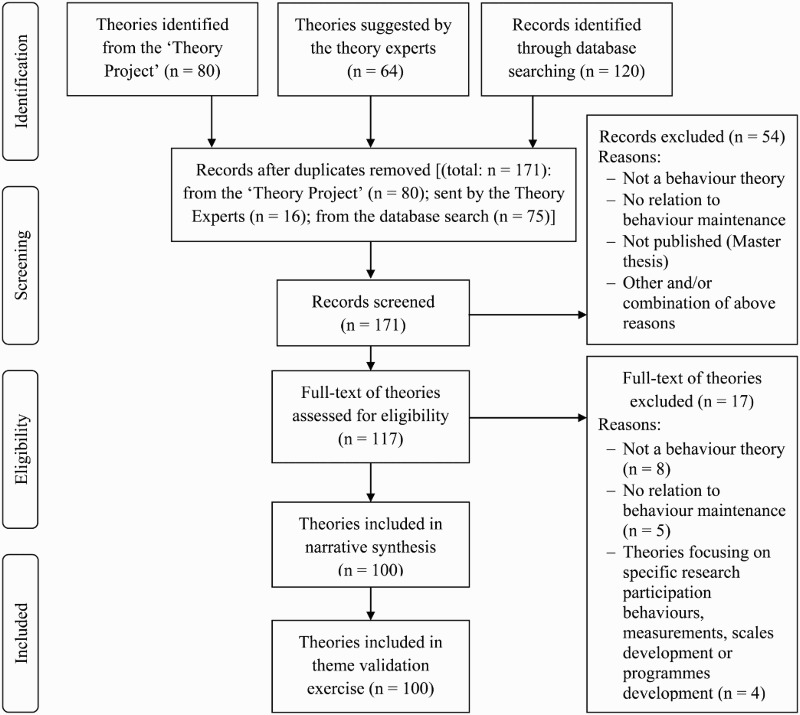
PRISMA 2009 flow diagram (Moher, Liberati, Tetzlaff, & Altman, 2009).

A total of 264 records were identified through the search strategy. After removal of duplicates, 171 records were screened for eligibility. Full texts of 117 theories (Appendix 2) were examined, out of which 100 published theories met the inclusion criteria (see Figure 2 for PRISMA diagram). To further validate the search, 40 international theory experts were contacted, out of them, 25 replied suggesting theories with relevant hypotheses for behaviour change maintenance. The most commonly suggested theories were the transtheoretical model of change (*n* = 11) (suggested by Prochaska & Di Clemente, 1983; Prochaska et al., 1992); the Health Action Process Approach (HAPA) (*n* = 10) (Schwarzer, 1992, 2008); social cognitive theory (*n* = 9) (Bandura, 1986); Marlatt's relapse prevention theory (*n* = 9) (Marlatt & George, 1984; Witkiewitz & Marlatt, 2004); Rothman's theory of maintenance (*n* = 8) (Rothman, 2000; Rothman, Baldwin, & Hertel, 2004; Rothman, Sheeran, & Wood, 2009); self-determination theory (*n* = 7) (Ryan & Deci, 2000) and habit theories (*n* = 6) (Verplanken & Aarts, 1999; Verplanken & Orbell, 2003) (Gardner, 2015).

Among the 100 included theories, 73 were behaviour specific (see Appendix 3). Fifty-three out of the 73 behaviour specific theories explained health-related behaviours. Most of the health behaviour specific theories built upon other existing theories and applied these to behaviour specific contexts. Twenty-six theories were behaviour specific *and* population specific; all population specific theories included in this review were also behaviour specific.

### How do current theories explain behaviour change maintenance?

Many behavioural theories do not explicitly address the issue of behavioural maintenance. Forty-three included theories involved assumptions of dynamic reciprocity, meaning assumptions that explanatory variables are modified through exposure to the target behaviour. For example, social cognition models, such as the theory of planned behaviour (Ajzen, 1991) hypothesise that behaviour is a function of cognitions about the desirability and controllability of behaviour. Repeated performance may lead to a re-evaluation of the behaviour and the individual may realise that it is less desirable or controllable than they thought when they adopted the behaviour. While many theories suggest that maintained behaviour is explained within the same theoretical constructs as behaviour initiation, the content (e.g., self-efficacy), direction and value of the constructs may change substantially from initiation to maintenance.

**Figure 3.**
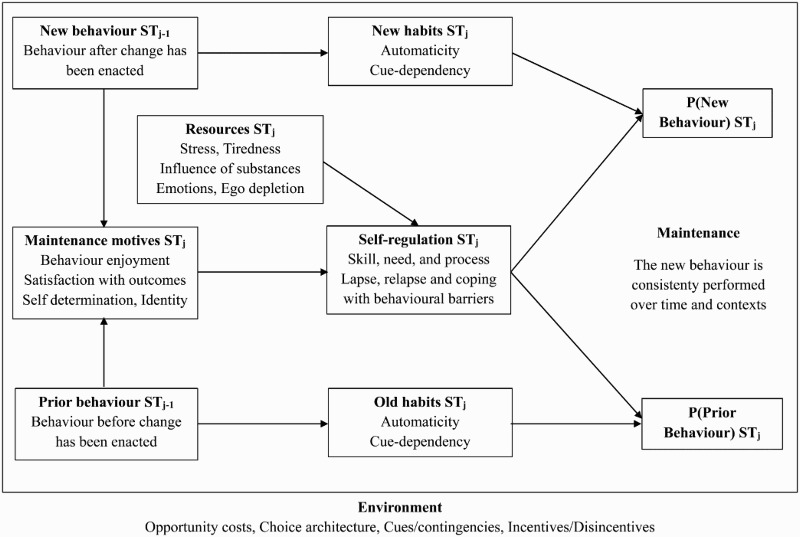
Hypothesised relationships between themes derived from maintenance theories in the process of behaviour change maintenance.Note: *ST_j_* describes the psychological situation S defined by features of the environment (e.g., available choices, external dis(incentives), cues and opportunity costs) and personal features (e.g., motivation, automaticity) at time point *T_j_*. New and prior behaviour are competing against each other after initial behaviour change.

For the remaining theories where differential explanatory hypotheses for initiation and maintenance could be derived, theory-guided thematic analysis resulted in five overarching interrelated themes (Table 2). These themes were cross-validated by 10 psychologists, whose independent assessments resulted in a high level of agreement (Krippendorff's *α* = 0.87). The five themes reflect specific theoretical explanations about how individuals maintain initial behaviour changes over time and in different contexts. Themes focus on the changing roles of motives, self-regulation, habits, resources and contextual factors from initial behaviour change to successful maintenance. Explanatory hypotheses for behaviour change maintenance are described for each theme, followed by specific examples taken from individual theories. Figure 3 provides an overview of the proposed inter-relationships between the five theoretical themes.

**Table 2.** Main theoretical themes relevant to maintenance identified in the review.

Theme	Brief theoretical explanation	Theories included (examples)
(1) Maintenance motives	People tend to maintain their behaviour if they have at least one sustained maintenance motive, i.e., they are satisfied with behavioural outcomes, they enjoy engaging in the behaviour; if behaviour is congruent with their identity, beliefs and values	Model of behaviour maintenance (Rothman, 2000) changed into a 2 × 2 behaviour change matrix (Rothman et al., 2009) Regulatory fit theory (Higgins, 2006) Self-determination theory (Deci & Ryan, 1985)
(2) Self-regulation	People tend to maintain behaviour if they successfully monitor and regulate the newly adopted behaviour and have effective strategies to overcome barriers to the performance of the new behaviour	Self-regulation theory (Kanfer & Gaelick, 1991) Relapse prevention theory (Marlatt & George, 1984) Dual process model of self-control (Hofmann et al., 2008)
(3) Resources	People are successful in maintaining behaviour if their psychological and physical resources are plentiful	Reflective and impulsive model (Strack & Deutsch, 2004) Self-control theory (Baumeister, 2002; Muraven & Baumeister, 2000) Goal conflict model (Stroebe, Mensink, Aarts, Schut, & Kruglanski, 2008)
(4) Habit	People are effective with maintaining behaviours which have become habitual and are supported by automatic responses to relevant cues	Health-related model of behaviour change (Hunt & Martin, 1988) Habit theory (Verplanken & Aarts, 1999; Verplanken & Orbell, 2003; Verplanken et al., 2008) Process model of lifestyle behaviour change (Greaves, Reddy, & Sheppard, 2010)
(5) Environmental and social influences	A supportive environment and social support are important for behaviour change maintenance. People tend to maintain behaviour which is in line with relevant social changes	Social cognitive/learning theory (Bandura, 1986) Social change theory (Thompson & Kinne, 1990) Normalisation process theory (May & Finch, 2009)

### Theme 1: Maintenance motives

Motives are the drivers for volitional behaviour. They help establishing priorities and allocating resources. Initial behaviour change is often motivated by expectations of uncertain long-term outcomes (e.g., changing one's diet to reduce health risks). It has been hypothesised that maintenance motives are often different from those motives that prompted individuals to make initial changes. Motives are hypothesised to be particularly facilitating for maintenance if they enable regular gratification from enacting the new behaviour, rather than from the experience of changing. For example, by focusing on behaviour enjoyment, satisfaction with behavioural outcomes, self-determination or if individuals experience congruence of the newly adopted behaviour with their identity, beliefs and values.

#### Enjoyment of behaviour and satisfaction with outcomes

Behaviour is more likely to be sustained if the reinforcement structure emphasises immediate and affective outcomes rather than long-term and rational outcomes. Motivation to avoid negative health consequences is hypothesised to be insufficient to maintain preventive behaviour that requires maintained effort. Therefore positive maintenance motives are needed (precaution adoption process model – Weinstein, 1988; Weinstein & Sandman, 1992). Individuals engage more strongly in what they are doing if they ‘feel right about it’ and if it fits with their decisions and prior engagement (regulatory fit theory – Higgins, 2006). This may include the enjoyment of performing the behaviour as such, or the enjoyment of immediate outcomes of the behaviour (temporal self-regulation theory – Hall & Fong, 2007; model of behaviour maintenance – Rothman, 2000; Rothman et al., 2004; Groningen active living model – Stevens, Bult, de Greef, Lemmink, & Rispens, 1999). After initial adoption of new behaviours individuals are theorised to evaluate the results of their efforts cognitively and emotionally. If the actual results reflect the desired results, the initial motivation is reinforced, and individuals are likely to make positive self-judgements and to sustain their efforts (behavioural model of medication adherence – De Bruin, Hosters, Van Den Borne, Kok, & Prins, 2005). The nature and timing of anticipated and experienced outcomes impacts on behaviour change maintenance.

#### Self-determination

Immediate changes in behaviour are often motivated by extrinsic motivation (i.e., an individual thinking that they ought to comply with external demands). However, intrinsic motivation (i.e., the individual wanting to maintain the new behaviour) is hypothesised to have a stronger influence on behaviour maintenance than extrinsic motivation (self-determination theory – Deci & Ryan, 2000; Ryan & Deci, 2000). Similarly, behaviour change is more likely to be maintained if a new behaviour resembles an individual's values and is perceived as personally relevant. Initially extrinsically motivated behaviour may develop intrinsic features over time and repeated performance.

#### Identity

Individuals are thought to be more likely to maintain behaviours which are in line with the beliefs they have about themselves (self-concept theory – Bracken, 1996; self-schema theory – Markus, 1977). Self-identity and nested beliefs and values can change as a result of engaging with a behaviour. For instance, an individual who exercises regularly can develop the self-representation of being a sportsperson or a person who no longer smokes can develop the identity of a non-smoker. Such changes in identity positively enhance behaviour change maintenance. These beliefs guide behaviour change maintenance as they lead and organise the processing of self-relevant information and standards, and create ongoing positive experiences associated with the new behaviour.

Initial behaviour change that is triggered by a significant life event or crisis can cause a shift in an individual's identity (process of reinvention theory – Epiphaniou & Ogden, 2010; Ogden & Hills, 2008). Such event-triggered changes are hypothesised to be particularly sustainable. A life event or crisis usually relates to relationships, health or other changes in circumstances judged to be highly significant. Behaviour change as a result of a life event is often maintained if the individual no longer perceives themselves to benefit from the prior behaviour, if there are fewer opportunities to perform the prior behaviour or if they believe that the prior behaviour was the cause of the crisis. Individuals experience shifts in their beliefs, followed by identity changes which support behaviour change maintenance (Epiphaniou & Ogden, 2010).

Congruence with identity is theorised as a key feature of behaviour internalisation processes, behavioural regulation and behaviour change maintenance (health behaviour internalisation model – Bellg, 2003). Internalisation is described as the learning of values or attitudes. Self-needs (i.e., identity, self-determination, security and support) and behaviour-related needs (i.e., preference, context competence and coping) interact and influence internalisation and self-regulation of the new health-related behaviour leading to behaviour change maintenance. Individuals are biased to self-regulate their behaviour in line with their self-concept.

### Theme 2. Self-regulation

Self-regulation refers to any effort to actively control behaviour by inhibiting dominant and automatic behaviours, urges, emotions or desires, and replacing those with goal-directed responses (Baumeister, Heatherton, & Tice, 1994). Theoretical explanations for behaviour change maintenance utilising self-regulation focus on the need for active behaviour control, on self-regulation skill, and the process of self-regulation and coping with barriers. Their relative contributions are dependent on personal resources, the strength of habits and environmental influences (Figure 3).

#### Self-regulation need

The need for active self-regulation changes over time and the course of the behaviour change process. Figure 1 illustrates how the behavioural potential (dominance of response) of the new behaviour and conflicting responses may vary over time as a result of individual and contextual factors. Individuals often initiate behaviour change when the moment is right, e.g., at times when opportunity costs and goal conflicts are low and motivation and capacity high. Context-specific cues, options and opportunity costs as well as varying levels of motivation may increase the risk of momentary lapses (Heckhausen & Beckmann, 1990; Rotter, 1960).

In particular, self-regulation is thought to be needed when new behaviours are not fully automatic and the behavioural potential of prior behaviours is still high (model of behaviour maintenance – Rothman, 2000; Rothman et al., 2004). Temporal changes in motivation to maintain behaviour change are directly related to self-regulation need; when motivation decreases, the need for active self-regulation increases. Some theorists hypothesise that the need for self-regulation decreases as individuals repeatedly engage in the new behaviour as they unlearn previous habits and develop new ones (habit theory – Verplanken & Aarts, 1999) and build self-efficacy to maintain behaviour (HAPA – Schwarzer, 1992, 2008). Conversely, some theoretical accounts suggest that the need for self-regulation may increase over time as repeated effort and self-restriction result in ego depletion (strength model of self-control – Baumeister, 2002, 2003; Baumeister & Heatherton, 1996) and decrease in motivation over time.

#### Self-regulation skill

Individuals differ in their skill to regulate their behaviour, known as ‘self-regulatory capacity’ (temporal self-regulation theory – Hall & Fong, 2007). Over time, costs and resources vary and the skill to successfully self-regulate behaviour is more important when self-regulatory tasks are more challenging. These stable differences in self-regulatory capacity are hypothesised to reflect executive control functions (e.g., planning skill, inhibition control, task switching) and they explain the ability to override automated responses (Nederkoorn, Houben, Hofmann, Roefs, & Jansen, 2010). Those with lower self-regulatory capacity are hypothesised to show weaker intention–behaviour relationships (Hall, Fong, Epp, & Elias, 2008). With time and repetition, individuals may develop better skills to actively self-regulate their behaviour (model of self-control – Muraven & Baumeister, 2000).

#### Self-regulation processes

Self-regulation includes the ongoing processes of self-monitoring, self-evaluation and self-reinforcement (Kanfer & Karoly, 1972) as well as dealing with temptations, hedonistic and impulsive influences that are in conflict with long-term goals. Behaviour change maintenance is theorised as an outcome of active and on-going self-regulation. The process of self-regulation is based on a system of hierarchically organised goals. Individuals monitor their behaviour against their goals and adjust their efforts if they perceive discrepancy between the current and a desired state (control theory – Carver & Scheier, 1982). If no discrepancy is detected or if behaviour exceeds standards, the individual will feel satisfied (self-regulation theory – Kanfer & Gaelick-Buys, 1991). Behaviour that falls short of relevant goals causes dissatisfaction and leads the individual to either spend more efforts or disengage from the goal.

#### Lapse, relapse and coping with behavioural barriers

Self-regulation involves coping with behavioural barriers, overcoming temptations, managing lapses and avoiding relapses. A lapse is a singular event in which an individual deviates from their desired goal (e.g., a cigarette smoked during an abstinence attempt). It may occur when the behavioural potential is high for the prior behaviour and when motivation to adhere to new health behaviour is low. A relapse is a sequence of lapses strung together, a setback, that causes reversion to the prior behaviour (e.g., regular smoking) (Witkiewitz & Marlatt, 2004). Relapse prevention theory includes variables that directly influence behaviour change maintenance, but not necessarily behaviour initiation, including interpersonal stress, cravings, mood and self-efficacy for managing lapses (Hendershot, Witkiewitz, George, & Marlatt, 2011).

In response to situations with a high risk of lapse or relapse, individuals may apply effective coping responses and as a result their self-efficacy and positive outcome expectancies increase and the probability of future relapse decreases (relapse prevention theory – Marlatt & George, 1984). Nevertheless, individuals may fail to apply effective coping responses in high risk situations and as a result their self-efficacy and positive outcome expectancies decrease; then individuals often return to their prior behaviour and their probability of future relapses increases (Marlatt & George, 1984).

In order to maintain a behaviour, an individual needs to develop high coping self-efficacy to overcome barriers (HAPA – Schwarzer, 1992, 2008). An individual with a high level of coping self-efficacy responds confidently to behavioural barriers with effective behaviour change maintenance strategies (i.e., action planning and coping planning), with sustained effort and prolonged persistence. Recovery self-efficacy, on the other hand, addresses the experience of failure followed by the recovery from a setback (HAPA – Schwarzer, 2008). Coping planning involves prospectively linking coping strategies to anticipated barriers to behaviour change (Sniehotta, Schwarzer, Scholz, & Schuz, 2005). Action planning is considered to be more important during behaviour initiation, whereas coping planning is hypothesised to facilitate behaviour change maintenance (Sniehotta et al., 2005).

### Theme 3. Habits

Habits are actions that have become automatically triggered by situational cues (Lally, Van Jaarsveld, Potts, & Wardle, 2010; Verplanken, 2006). Habit development follows a period of successful self-regulation of a new behaviour. Consciously controlled behaviours become automatic over time with repetition and are theorised to be performed outside of conscious awareness (Hofmann, Friese, & Wiers, 2008; Hunt & Martin, 1988; Rothman et al., 2009; Verplanken & Aarts, 1999). Developing new favourable, habitual cue responses can help maintain new behaviours, however the stronger prior habits are, the more likely an individual is to lapse to the prior behaviour. Removing cues that may trigger unhealthy behaviour from the environment supports behaviour change maintenance by means of stimulus control (transtheoretical model of change – Prochaska & Di Clemente, 1983; Prochaska et al., 1992).

#### Dual process models and habit theories

In dual process models behaviour is theorised to be governed by the interplay of two regulatory systems, a reflective system, which is based on conscious deliberation, controlled but effortful, which aims to override a quicker automatic system that responds in line with habits and impulses (dual-system models – Strack & Deutsch, 2004). Individuals can maintain behaviour through ongoing self-regulation, however, self-regulation draws on finite psychological resources which can be depleted. When resources are low self-regulation is likely to fail. Thus, the most sustainable mechanism for maintenance is to develop automaticity for the newly adopted behaviour.

Behaviour is a function of reflective and automatic processes (health-related model of behaviour change – Hunt & Martin, 1988; 2 × 2 behaviour change matrix – Rothman et al., 2009; theory of interpersonal behaviour – Triandis, 1977). Higher levels of cognitive processing are usually not necessary when activities become habitual. Habitual behaviours are carried out with minimum awareness while conscious behaviours are performed only occasionally. Relegation of habits to lower levels of consciousness is seen as part of the human adaptation process. Individuals are able to deal with a large number of internal and external stimuli because habitual behaviours require minimum levels of awareness and resources. Higher level self-regulation, decision-making and monitoring processes can then deal with more novel features of the environment. Habit facilitates behaviour change maintenance as desired activities become habitual, taking place outside of conscious awareness (habit theory – Verplanken & Aarts, 1999). Reflective and automatic influences are altered by boundary conditions (i.e., habitualness, ego depletion (impulsive versus reflective framework) Hofmann et al., 2008).

#### Learning theories and habit

Learning theories provide explanations for the influence of the environment on behaviour and for the development of habits. Repetition and reinforcement are considered as a key to habit formation (and associative theories of goal-directed behaviour – de Wit & Dickinson, 2009; Pavlovian instrumental transfer – Hall, Parkinson, Connor, Dickinson, & Everitt, 2001; classical conditioning – Pavlov, 1927 and operant conditioning – Skinner, 1953; two-factor avoidance theory – Stasiewicz & Maisto, 1993). Behaviour change maintenance is promoted by a number of factors such as situating new learning in the most relevant contexts, providing retrieval cues after the new learning is complete, and varying the contexts in which the new learning takes place (a learning theory perspective on the maintenance of behaviour change – Bouton, 2000). Learning theories provide a wide range of explanations for how new behaviours are acquired and become habitual.

In order to maintain behaviour it is hypothesised that it is beneficial for individuals to be conditioned to certain behavioural responses occurring in a given situational context. For new conditioned reflexes to be established any external stimulus (which will become the signal in a conditioned reflex) must overlap in time with the action of an unconditioned stimulus (classical conditioning – Pavlov, 1927). After repeated association of a stimulus with a behavioural response, maintenance of behaviour develops. Individuals can be conditioned to perform new behaviours if a stimulus and its behavioural response become associated; to break an existing association, stimuli and response must be disassociated (classical conditioning – Pavlov, 1927; operant conditioning – Skinner, 1953). For instance when individuals adopt a new, healthy diet, they are conditioned to certain stimuli (e.g., lunch time), which produces a situational response (e.g., consuming a salad). Repeated association of stimuli and response leads to behaviour change maintenance. Unleaning or disassociation is a slow process and in some cases does not occur for behaviours which are engrained and thus still remain response options even after many years of not practicing them.

### Theme 4. Resources

Resources are psychological and physical assets that can be drawn on during the process of behavioural regulation. As maintaining behaviour change is often difficult and requires sustained efforts, resources available to the individual are hypothesised to play an important role. When resources are limited or depleted due to stress, tiredness, exhaustion and intoxication, an individual's self-regulatory capacity is limited.

Individuals are more likely to initiate behaviour change at times when their psychological and physical resources are plentiful and when opportunity costs (effort needed to enact behaviour) are low (i.e., resources are not immediately needed for competing demands). Over time opportunity costs and resources vary and habitual behaviours are likely to dominate when resources are limited (see Figure 1, situation B). The processes of breaking old habits and developing new ones are likely to act in conjunction for behaviour change maintenance. Lapse and relapse are thus, theorised to be associated with low levels of resources.

Dual process models offer a perspective on understanding how resources impact on behaviour change maintenance. It is hypothesised that behaviour can be governed by either a resource-intensive reflective system or a mostly automated impulsive system and that resources act as moderators (boundary conditions) determining which of the system generates the response (dual-system models (impulsive versus reflective framework) Hofmann et al., 2008; dual-system models – Strack & Deutsch, 2004). Even when cognitive resources are limited effective maintenance can occur if the behaviour is habitually instigated.

#### Self-regulation as a limited resource

It is suggested that self-regulation draws on finite mental resources which become depleted through the use of self-regulatory processes and take time and to rest and recover (strength model of self-control – Baumeister, 2003; Hagger, Wood, Stiff, & Chatzisarantis, 2009; Muraven & Baumeister, 2000). Coping with stress, resisting temptations, and controlling emotions requires additional self-regulatory efforts and each additional attempt to self-regulate is more likely to fail. When cognitive resources are limited, individuals who are actively self-regulating to engage in a new behaviour are prone to engaging in impulsive or automatic actions which reflect the prior behaviour (strength model of self-control – Baumeister, 2002, 2003; Baumeister & Heatherton, 1996). For instance, dieters are vulnerable to uncontrolled eating when cognitive processes are disrupted (dietary restraint theory – Polivy & Herman, 1985). Self-regulation or willpower draw upon a mental resource and require energy which can be depleted; individuals who actively self-regulate are less effective at subsequent tasks that also require self-control (Hagger et al., 2013). Rest and positive affect help restore these resources (Baumeister, 2003).

#### Inter-individual differences in resources and resources availability

There are inter-individual differences in the availability of cognitive resources required to override automatic responses (dual-system models – Strack & Deutsch, 2004). When resources are in short supply, the automatic system overrides the reflective one, often leading to maintenance failure. Dispositional or situational moderators shift the weight between impulsive and reflective influences (impulsive versus reflective framework – Hofmann et al., 2008). Restrained cognitive resources paired with unconscious positive expectations towards unhealthy behaviour may hinder behaviour change maintenance. Conditions that have been hypothesised to also hinder behaviour change maintenance, include ego depletion (Collins & Lapp, 1992), high cognitive load (Friese, Hofmann, & Wanke, 2008), low working memory capacity (Fletcher, Marks, & Hine, 2011), and the influence of alcohol and other substances (Gunn, Finn, Endres, Gerst, & Spinola, 2013).

Throughout the lifespan, social and individual resources change and individuals develop, elaborate and commit to individual goals (model of selection, optimisation and compensation – Baltes, 1997). Goal selection can be elective or in response to experienced change in resources. During elective goal selection individuals define their goals in order to match their individual needs and motives. Loss-based goal selection is a response to the loss of previously available resources. Optimisation refers to acquiring, applying and refining goal-directed means. Individuals adjust their goals to focus or redirect their efforts to maintain functioning or they substitute for a functional loss via compensation.

### Theme 5. Environment and social influences

The final theme includes features of the social and environmental context of action which provide available options, sets incentives and disincentives, opportunity costs and cues with contingency for behavioural responses. This theme includes aspects of the choice architecture that alter the opportunity costs and accessibility of behaviour options (Thaler & Sunstein, 2008). Many theories emphasise the role of a supportive external environment on behaviour change maintenance, and most ecological models of behaviour suggest equal explanations for behaviour initiation and maintenance (Bronfenbrenner, 1977, 1986; McLeroy, Bibeau, Steckler, & Glanz, 1988; Panter-Brick, Clarke, Lomas, Pinder, & Lindsay, 2006). However, some theories such as habit theories (Hofmann et al., 2008; Hunt & Martin, 1988; Rothman et al., 2009; Verplanken & Aarts, 1999), hypothesise that environmental cues related to prior and newly adopted behaviours explain whether or not a newly adopted behaviour is maintained. A supportive environment, positive social influences and constructive social change facilitate maintenance of behaviour change as it lowers the opportunity costs of the new behaviour. Environmental factors determine the amount of active self-regulation and resource required by the individual and behaviour change is less likely to be maintained in environments which are not conducive of the newly adopted behaviour (Mackenbach et al., 2014).

#### Environment

As active self-regulation is effortful, the default mode is to respond to the behavioural option most facilitated by the environmental choice architecture (Figure 1). Individuals may develop context-dependent associations which are easy to maintain in the same environment (process model for supporting lifestyle behaviour change – Greaves et al., 2010; impulsive versus reflective framework – Hofmann et al., 2008; 2 × 2 behaviour change matrix – Rothman et al., 2009; habit theory – Verplanken & Aarts, 1999). It is theorised that if individuals change their context, they are more likely to perform non-habitual behaviour (habit theory – Verplanken, Walker, Davis, & Jurasek, 2008). When the environment changes, a window of opportunity opens for habit change (habit theory – Verplanken et al., 2008) and individuals may be more likely to maintain habitual behaviours in stable contexts. Environmental changes can present a threat for habitual behaviours but also opportunity for new behaviours.

#### Social influence

Like environmental factors, social influence affects the opportunity costs and incentive structure for behavioural options in a given context. It can affect the effort needed to perform new behaviours. Provided support can increase individual capacity to maintain behaviour (e.g., by providing encouragement or help). Social influence occurs when an individual's opinions, emotional states and behaviours are affected by others. Knowledge and skills are acquired through social modelling; the observation and replication of other peoples’ actions (social cognitive theory – Bandura, 1986).

Individuals are more likely to follow the guidance given to them by people they trust and they feel connected to (Bandura, 1986). Thus, to maintain a new behaviour a sense of relatedness must be developed (self-determination theory – Deci & Ryan, 2000; Ryan & Deci, 2000). Group membership define individuals; individuals are motivated to evaluate their group positively, usually showing a preference for a group they belong to as compared to other groups (social identity theory – Turner, 1987). Individuals attempt to achieve or to maintain positive social identity (social identity model – Tajfel & Turner, 1979). They sustain actions which are in line with group norms and which are approved by group members (e.g., substance abuse theory – Neff & MacMaster, 2005). Thus, individuals tend to follow the social norms and rules of the group they belong to.

#### Social change – how norms are shaped, accepted and maintained

Social change can facilitate behaviour change maintenance. Large-scale behavioural change is often achieved by changing the standards of what is acceptable in a given community (social change theory – Thompson & Kinne, 1990). Social change is described as a three step process: unfreezing, moving and freezing (theory of change – Lewin, 1951) or implementation, embedding and sustaining (normalisation process theory – May & Finch, 2009; May et al., 2009). The process of freezing is similar to maintaining newly introduced behaviours, which have become social norms and which are accepted in the given context (theory of change – Lewin, 1951). ‘Social habits’ are socially accepted behaviours that are resistant to change; ‘a quasi-stationary equilibrium state’ is stable and susceptible to change state which at the same time is underpinned by an on-going social process (theory of change – Lewin, 1951). Sustaining embedded practices in their social contexts, also called ‘integration’, is relevant to maintaining behaviours (normalisation process theory – May & Finch, 2009; May et al., 2009). Through social change, practice can become routinely embedded in everyday life. For social change to be maintained, individuals should feel responsible for the programmes promoting change and they should take control over them so they continue to maintain them after initial organising efforts (social change theory – Thompson & Kinne, 1990).

## Discussion

### Main findings

By systematically reviewing maintenance-relevant behaviour change theories, five overarching interconnected theoretical themes emerged: maintenance motives, self-regulation, resources, habits and contextual influences. Based on these themes, differential hypotheses for the initiation and maintenance of health-related behaviours have been formulated. In Figure 3 a graphical illustration of the review findings is presented, integrating these theoretical themes, and summarising current theoretical concepts relevant for behaviour change maintenance.

Individuals need at least one sustained motivator to maintain behaviour; these may include behaviour enjoyment, satisfaction with behavioural outcomes, self-determination or an experience of behavioural congruence with beliefs and values, all of which often develop after initiating a new behaviour. It is likely that individuals start behaviour change attempts at times when their motivation is at the highest and opportunity costs are low (Figure 1). As motivation and opportunity costs regress to the mean (i.e., as motivation decreases and costs increase), the need for self-regulatory effort is increased in order to ensure that the new behaviour continues despite less than optimal conditions.

The ongoing and active use of limited cognitive self-regulatory resources can result in ego depletion, with stress, tiredness, substance use and negative affect also leading to a decreased ability to exert control over, or self-regulate, behaviour. At different times and depending on the availability of cognitive resources, motivation and level of depletion, behaviour change maintenance may alternate between needing to be actively self-regulated and being automatic, context-driven and effortless. With repeated performance of a new behaviour, the need for conscious self-regulation decreases and behaviour becomes habitual, which in turn increases the chance that it will be maintained.

Behaviour, whether under conscious control or occurring automatically and habitually, occurs within an environmental and social context, with such influences serving to either facilitate or hinder behaviour change maintenance. As with the initiation of behaviour change, stable contexts make behaviour and habits easier to sustain. Thus, ecological factors are important for both behaviour initiation and maintenance.

### Comparison with other studies

This systematic theory review adds to existing knowledge by providing a comprehensive review of theories to identify theoretical hypotheses about maintained behaviour change. While previous reviews have focused on behaviour change (Cane, O'Connor, & Michie, 2012; Michie et al., 2005), this is the first review to specifically assess theoretical explanations for behaviour change maintenance. Overall, this review provides a summary of theoretical explanations for behaviour change maintenance; however, further research is needed to test the proposed relationships within and between the emerging themes.

### Strengths and limitations

The main strength of this review is the comprehensive and rigorous search and the thematic theoretical synthesis of available theories addressing behaviour change maintenance. In this study, a new approach to theory analysis was presented, including identification of theoretical explanations and theory synthesis specifically in relation to behaviour change maintenance. This review not only identified themes that relate to behaviour change maintenance, but also resulted in the formulation of a number of theoretical predictions including the inter-relationships and dependencies between themes.

While the resulting summary provides a detailed description of broad themes and relationships relevant to maintenance, for a more detailed account of specific predictions, individual theories may be more relevant. In the validation exercise, experts were asked to assign theoretical statements to the themes emerging from the synthesis in this review. It cannot be ruled out, that a different set of themes would have represented the data equally well or better.

This theory review drew mainly on theories which were designed and/or assessed in the context of health-related behaviours, and while the findings may be applicable to other contexts and behaviours, the degree of generalisability requires testing. While some theories have been tested (Hagger et al., 2009; Kwasnicka, Presseau, White, & Sniehotta, 2013; McEachan, Conner, Taylor, & Lawton, 2011) many theories lack systematic empirical evaluations. Similarly, the theoretical themes that emerged in this review will require further evaluation, development and testing as part of a systematic interative process of theory building (Head & Noar, 2014; Noar & Head, 2014; Rhodes, 2014; Sniehotta, Presseau, & Araujo-Soares, 2015).

### Unanswered questions and future research

The main contribution of this review is the identification of the main hypotheses for behaviour change maintenance formulated in the theoretical literature to date. It provides a platform for future research and practice in behaviour change maintenance, which is a key priority area in behaviour change science. Future research should systematically review the existing evidence for each theoretical theme; and undertake further empirical testing of each explanation, followed by empirical testing of an integrated theoretical model. Evidence from theory-based interventions should be used to revise, refine or reject theoretical principles (Rothman, 2004). Dialogue between theory and practice is encouraged so that theories are not only used to design interventions, but interventions also inform the redevelopment of theories. Predictions and theoretical explanations should be tested across a variety of settings and populations, facilitating theory development. Future research should focus on the informed development of behaviour change maintenance theory, test themes presented within this review, explore relationships between themes and constructs, and add new theoretical predictions.

### Implications for health behaviour change maintenance and conclusions

The theoretical explanations described within the five overarching themes can be applied in health contexts to help explain maintenance of health-related behaviours. Listed theoretical assumptions may be applied to facilitate interventions targeting health promotion and maintenance of health behaviours. They can serve as guidance for intervention developers who can target five maintenance processes:

1. Helping individuals to maintain positive behaviour change maintenance motives, emphasising positive outcomes of a new health behaviour, providing behavioural options which are enjoyable, inspiring individuals to redefine themselves in line with new healthy lifestyle principles.
2. Facilitating behaviour self-regulation; for instance through self-monitoring behaviour and helping individuals to develop effective strategies to overcome behavioural barriers and to prevent relapse.
3. Facilitating habit development and maintenance for positive health behaviour changes; for instance by reshaping the environment and making healthy options salient and by cuing individuals towards healthy behaviours.
4. Providing individuals with resources that are needed to successfully maintain a new health behaviour. Resources can be physical (e.g., sport facilities, health products) or psychological (e.g., self-regulation training, mindfulness and relaxation methods).
5. Reshaping the environment at individual, social and community levels. Providing social support and introducing social changes that are in line with positive health behaviour change maintenance.

Specific theoretical explanations of behaviour change maintenance have been presented in this review. This review examined and summarised 100 theories that explicitly or implicitly explain how individuals maintain new behaviours. Five theoretical themes included maintained motivation, active self-regulation, habitual cue driven responses and boundary conditions including resources and environmental factors. This review can act as a starting point on the journey towards an integrated behaviour change maintenance theory.

Supplemental_Data

## Note

1. The list of theories was based on one received on 9 February 2012 by the Theory Project Team (final list of theories published here: Michie et al., 2014).
