# Supplementary material for: Theoretical explanations for maintenance of behaviour change: a systematic review of behaviour theories
Source: Health Psychol Rev. 2016 Mar 7;10(3):277–96. doi: 10.1080/17437199.2016.1151372 (PMC4975085; doi:10.1080/17437199.2016.1151372)
Supplement: Supplemental_Data [file rhpr_a_1151372_sm2761.pdf]

Appendix 1. Full search strategy (On-line supplement)

Full search strategy was informed by the Theory Project search strategy (Michie, Campbell, Brown, & West, 2014). The strategy replicated the Theory Project search (sets 1-3) adding maintenance specific terms (set 4), the results of the search were overlapped with results of the Theory Project search results and only additional new hits identified after adding maintenance-relevant items were screened (120 relevant records).

Search terms and structure was consistent across selected databases; limits were set in line with the exclusion criteria. Four sets of terms were used representing: set 1) theory terms which have only or predominantly been applied to changing behaviour; set 2) theory terms relevant to behaviour change but which have also been otherwise applied as theoretical tools to understanding behaviour more generally; set 3) change terms; set 4) maintenance relevant terms. The search string for the original electronic search was run across the three databases as follows:

Set 1: Behaviour change theory terms: “behavior change theories” OR “behavior change theory” OR “behavior theories” OR “behaviour change theories” OR “behaviour change theory” OR “behaviour theories” OR “diffusion of innovations” OR “elaboration likelihood model” OR “goal theory” OR “information motivation behavioral skills model” OR “information motivation behavioural skills model” OR “rational addiction model” OR “social cognition models”

Set 2: Behaviour theory terms: “acculturation theory” OR “AIDS risk reduction model” OR “behavior economic theories” OR “behaviour economic theories” OR “communication theory” OR “community organisation theory” OR “community organization theory” OR “consumer information processing model” OR “control theory” OR “critical consciousness” OR “decisional balance theory” OR “ecological model” OR “ecological perspective” OR

"empowerment theory" OR "enculturation theory" OR "exchange theory" OR "fear arousal theory" OR "goal setting theory" OR "goal theory" OR "habit theory" OR "health belief model" OR "health promotion theories" OR "health promotion theory" OR "health behaviour theory" OR "health behavior theory" OR "innovation-decision process" OR "interactionist model" OR "intrapersonal theory" OR "intrinsic motivation theories" OR "multicomponent stage model" OR "natural recovery" OR "operant learning theory" OR "operant theory" OR "organisational change theory" OR "organizational change theory" OR "personality theory" OR "precaution adoption process" OR "protection motivation theory" OR "reciprocal causality" OR "reciprocal determinism" OR "risk behaviour theory" OR "risk behavior theory" OR "self regulation theory" OR "self-regulation theory" OR "self-determination theory" OR "self-efficacy theory" OR "self-perception theory" OR "social capital" OR "social cognitive theory" OR "social comparison theory" OR "social determinism" OR "social influence" OR "social learning theories" OR "social learning theory" OR "social marketing theory" OR "social structural theory" OR "social support" OR "stage of change model" OR "stages of change model" OR "systems theory" OR "theories of planned behavior" OR "theories of planned behaviour" OR "theory of planned behavior" OR "theory of planned behaviour" OR "theory of reasoned action" OR "transtheoretical model" OR "value-expectancy theory"

Set 3: Change terms: "behaviour change" OR "behavior change" OR "behaviour modification" OR "behavior modification" OR "mediation effects on behaviour" OR "mediation effects on behavior" OR "normative change" OR "normative changes" OR "cultural change" OR "cultural changes" OR "social change" OR "social changes" OR "group level effect" OR "group level effects" OR "social development" or "social developments" OR "behavioural interventions" OR "behavioral interventions" OR "behavioural intervention" OR "behavioral intervention".

Set 4: Maintenance relevant terms: “maintenance” OR “behaviour maintenance” OR “behavior maintenance” OR “maintain” OR “sustain” OR “sustained behaviour” OR “sustained behavior” OR “sustained change” OR “habit” OR “habitual behaviour” OR “habitual behavior” OR “maintenance stage”

Appendix 2. Full text references assessed for inclusion in the Theory Review

Ajzen, I. (1985). *From intentions to actions: A theory of planned behavior*: Springer.

Alfonzo, M. A. (2005). To walk or not to walk? The hierarchy of walking needs. *Environment and Behavior, 37*(6), 808-836.

Antonovsky, A. (1996). The salutogenic model as a theory to guide health promotion. *Health promotion international, 11*(1), 11-18.

Bagozzi, R. P. (1992). The self-regulation of attitudes, intentions and behavior. *Social Psychology Quarterly, 55*(2), 178-204.

Balleine, B. W., & Killcross, S. (2006). Pavlovian–instrumental transfer. *Trends in Neurosciences, 29*(5), 272-279.

Baltes, P. B. (1997). On the incomplete architecture of human ontogeny - Selection, optimization, and compensation as foundation of developmental theory. *American Psychologist, 52*(4), 366-380. doi:10.1037//0003-066x.52.4.366

Bandura, A. (1977). Self-efficacy: toward a unifying theory of behavioral change. *Psychological Review, 84*(2), 191.

Bandura, A. (1986). Social Foundations of Thought and Action: A Social Cognitive Theory. Englewood Cliffs, NJ: Prentice-Hall.

Baumeister, R. F., Bratslavsky, E., Muraven, M., & Tice, D. M. (1998). Ego depletion: Is the active self a limited resource? *Journal of Personality and Social Psychology, 74*(5), 1252-1265. doi:10.1037/0022-3514.74.5.1252

Baumeister, R. F., Muraven, M., & Tice, D. M. (2000). Ego depletion: A resource model of volition, self-regulation, and controlled processing. *Social Cognition, 18*(2), 130-150. doi:10.1521/soco.2000.18.2.130

Becker, G. S., & Murphy, K. M. (1988). A theory of rational addiction. *The Journal of Political Economy*, 675-700.

Bellg, A. J. (2003). Maintenance of health behavior change in preventive cardiology - Internalization and self-regulation of new behaviors. *Behavior Modification, 27*(1), 103-131. doi:10.1177/0145445502238696

Bem, D. J. (1967). Self-perception: An alternative interpretation of cognitive dissonance phenomena. *Psychological Review, 74*(3), 183.

Bem, D. J. (1972). Self-perception theory. *Advances in experimental social psychology, 6*, 1-62.

Berkowitz, A. D. (2004). The social norms approach: Theory, research and annotated bibliography. *Higher Education Center for Alcohol and Other Drug Abuse and Violence Prevention. US Department of Education*.

Boekaerts, M. (1997). Self-regulated learning: A new concept embraced by researchers, policy makers, educators, teachers, and students. *Learning and instruction, 7*(2), 161-186.

Bouton, M. E. (2000). A learning theory perspective on lapse, relapse, and the maintenance of behavior change. *Health Psychology, 19*(1), 57-63. doi:10.1037/0278-6133.19.Suppl1.57

Bowen, M. (1966). The use of family theory in clinical practice. *Comprehensive Psychiatry, 7*(5), 345–374.

Bracken, B. A. (1996). *Handbook of self-concept: Developmental, social, and clinical considerations*: John Wiley & Sons.

Bronfenbrenner, U. (1977). Toward an experimental ecology of human development. *American Psychologist, 32*, 513-531.

Bronfenbrenner, U. (1986). Ecology of the Family as a Context for Human Development: Research Perspectives. *Developmental Psychology, 22*(6), 723-742.

Burnet, D., Plaut, A., Courtney, R., & Chin, M. H. (2002). A practical model for preventing type 2 diabetes in minority youth. *The Diabetes Educator, 28*(5), 779-795.

Carver, C. S., & Scheier, M. F. (1982). Control theory: A useful conceptual framework for personality-social, clinical, and health psychology. *Psychological Bulletin, 92*, 111-135.

Catania, J. A., Kegeles, S. M., & Coates, T. J. (1990). Towards an understanding of risk behavior: An AIDS risk reduction model (ARRM). *Health Education & Behavior, 17*(1), 53-72.

Corbit, L. H., & Balleine, B. W. (2005). Double dissociation of basolateral and central amygdala lesions on the general and outcome-specific forms of pavlovian-instrumental transfer. *The Journal of neuroscience, 25*(4), 962-970.

Cummings, T., & Worley, C. (2014). *Organization development and change*: Cengage Learning.

Davis, F. D. (1989). Perceived usefulness, perceived ease of use, and user acceptance of information technology. *MIS quarterly*, 319-340.

De Bruin, M., Hosters, H. J., Van Den Borne, H. W., Kok, G., & Prins, J. M. (2005). Theory- and Evidence-Based Intervention to Improve Adherence to Antiretroviral Therapy Among HIV-Infected Patients in The Netherlands: A Pilot Study. *AIDS PATIENT CARE and STDs, 19*(6), 384-394.

De Vries, H., Mudde, A., Dijkstra, A., Norman, P., Abraham, C., & Conner, M. (2000). The attitude-social influence-efficacy model applied to the prediction of motivational transitions in the process of smoking cessation. *Understanding and Changing Health Behavior: From Health Beliefs to Self Regulation. Amsterdam, The Netherlands: Harwood Academic*, 165-187.

De Vries, H. d., Mesters, I., Steeg, H. v. d., & Honing, C. (2005). The general public’s information needs and perceptions regarding hereditary cancer: an application of the Integrated Change Model. *Patient education and counseling, 56*(2), 154-165.

de Wit, S., & Dickinson, A. (2009). Associative theories of goal-directed behaviour: a case for animal-human translational models. *Psychological Research-Psychologische Forschung, 73*(4), 463-476. doi:10.1007/s00426-009-0230-6

Deci, E. L., & Ryan, R. M. (2002). *Handbook of self-determination research*: University Rochester Press.

Deci, E. L., & Ryan, R. M. (2010). *Self-Determination*: Wiley Online Library.

DiMatteo, M. R., Haskard-Zolnierek, K. B., & Martin, L. R. (2012). Improving patient adherence: a three-factor model to guide practice. *Health Psychology Review, 6*(1), 74-91.

Dresler-Hawke, E., & Veer, E. (2006). Making healthy eating messages more effective: combining integrated marketing communication with the behaviour ecological model. *International Journal of Consumer Studies, 30*(4), 318-326.

Epiphaniou, E., & Ogden, J. (2010). Successful Weight Loss Maintenance and a Shift in Identity From Restriction to a New Liberated Self. *Journal of Health Psychology, 15*(6), 887-896. doi:10.1177/1359105309358115

Ewart, C. K. (1991). Social action theory for a public health psychology. *American Psychologist, 46*(9), 931.

Fishbein, M. (2000). The role of theory in HIV prevention. *AIDS care, 12*(3), 273-278.

Fishbein, M., & Ajzen, I. (1975). *Belief, attitude, intention and behavior: An introduction to theory and research*.

Fishburn, P. C. (1968). Utility theory. *Management science, 14*(5), 335-378.

Fisher, J. D., Fisher, W. A., Williams, S. S., & Malloy, T. E. (1994). Empirical tests of an information-motivation-behavioral skills model of AIDS-preventive behavior with gay men and heterosexual university students. *Health Psychology, 13*(3), 238.

Flay, B. R., d'Avernas, J. R., Best, J. A., Kersell, M. W., & Ryan, K. B. (1983). Cigarette smoking: Why young people do it and ways of preventing it. *Pediatric and adolescent behavioral medicine, 10*, 132-183.

Flay, B. R., DiTecco, D., & Schlegel, R. P. (1980). Mass media in health promotion: An analysis using an extended information-processing model. *Health Education & Behavior, 7*(2), 127-147.

Forgas, J. P. (1995). Mood and judgment: the affect infusion model (AIM). *Psychological Bulletin, 117*(1), 39.

Forgas, J. P. (2001). The Affect Infusion Model (AIM): An integrative theory of mood effects on cognition and judgments.

Friese, M., Hofmann, W., & Wanke, M. (2008). When impulses take over: Moderated predictive validity of explicit and implicit attitude measures in predicting food choice and consumption behaviour. *British Journal of Social Psychology, 47*, 397-419. doi:10.1348/014466607x241540

Gatersleben, B., & Vlek, C. (1998). Household consumption, quality of life, and environmental impacts: A psychological perspective and empirical study. *Green households*, 141-183.

Gebhardt, W. A. (1997). *Health Behaviour Goal Model: Towards a Theoretical Framework for Health Behaviour Change.* Leiden Leiden University Press.

Gerrard, M., Gibbons, F. X., Houlihan, A. E., Stock, M. L., & Pomery, E. A. (2008). A dual-process approach to health risk decision making: The prototype willingness model. *Developmental Review, 28*(1), 29-61.

Glasgow, R. E., Vogt, T. M., & Boles, S. M. (1999). Evaluating the public health impact of health promotion interventions: the RE-AIM framework. *American Journal of Public Health, 89*(9), 1322-1327.

Gonzalez, G. M. (1989). An integrated theoretical model for alcohol and other drug abuse prevention on the college campus. *Journal of College Student Development*.

Graybiel, A. M. (1995). Building action repertoires: Memory and learning functions of the basal ganglia. *Current Opinion in Neurobiology, 5*(6), 733-741. doi:10.1016/0959-4388(95)80100-6

Graybiel, A. M. (2005). The basal ganglia: learning new tricks and loving it. *Current Opinion in Neurobiology, 15*(6), 638-644. doi:10.1016/j.conb.2005.10.006

Greaves, C., Reddy, P., & Sheppard, K. (2010). Supporting Behaviour Change for Diabetes Prevention. In P. Schwarz, P. Reddy, C. Greaves, J. Dunbar, & S. J. (Eds.), *Diabetes prevention in practice* (pp. 19-29). Dresden: Tumaini Institute for Prevention Management.

Grossman, M. (1972). On the concept of health capital and the demand for health. *The Journal of Political Economy*, 223-255.

Hagger, M. S., Chatzisarantis, N. L. D., Culverhouse, T., & Biddle, S. J. H. (2003). The processes by which perceived autonomy support in physical education promotes leisure-time physical activity intentions and behavior: a trans-contextual model. *Journal of Educational Psychology, 95*(4), 784.

Hagger, M. S., Wood, C., Stiff, C., & Chatzisarantis, N. L. D. (2009). The strength model of self-regulation failure and health-related behaviour. *Health Psychology Review, 3*(2), 208-238.

Hall, P. A., & Fong, G. T. (2007). Temporal self-regulation theory: A model for individual health behavior. *Health Psychology Review, 1*(1), 6-52.

Hawkins, J. D., & Weis, J. G. (1985). The social development model: An integrated approach to delinquency prevention. *Journal of Primary Prevention, 6*(2), 73-97.

Heckathorn, D. D. (1990). Collective sanctions and compliance norms: A formal theory of group-mediated social control. *American Sociological Review*, 366-384.

Heckhausen, J. (2007). The motivation-volition divide and its resolution in action-phase models of developmental regulation. *Research in Human Development, 4*(3-4), 163-180.

Higgins, E. T. (2006). Value from regulatory fit. *Current Directions in Psychological Science, 14*(4), 209-213

Hofmann, W., Friese, M., & Wiers, R. W. (2008). Impulsive versus reflective influences on health behavior: a theoretical framework and empirical review. *Health Psychology Review, 2*(2), 111-137

Hoss, L. G. (1985). *Intrinsic motivation and cognitive evaluation theory: application to counseling and maintenance of behavior change.* State University of New York at Albany, Department of Counseling Psychology and Student Development.

Hunt, S. M., & Martin, C. J. (1988). Health-related behavioural change–A test of a new model. . *Psychology and Health, 2*, 209-230.

Jessor, R., & Jessor, S. (1977). *Problem behavior and psychosocial development: A longitudinal study of youth*. New York: Academic Press.

Jones, E. E. (1976). How Do People Perceive the Causes of Behavior? Experiments based on attribution theory offer some insights into how actors and observers differ in viewing the causal structure of their social world. *American Scientist*, 300-305.

Kanfer, F. H., & Gaelick-Buys. (1991). Self-management methods. In F. H. Kanfer & A. P. Goldstein (Eds.), *Helping people change: A textbook of methods* (pp. 305-360). New York: Pergamon Press.

Karasek, R. A. (1979). Job demands, job decision latitude, and mental strain: Implications for job redesign. *Administrative science quarterly*, 285-308.

Katz, D. L. (2001). Behavior modification in primary care: the pressure system model. *Preventive Medicine, 32*(1), 66-72.

Kielhofner, G. (2002). *A model of human occupation: Theory and application*: Lippincott Williams & Wilkins.

Kremers, S. P. J., De Bruijn, G.-J., Visscher, T. L. S., Van Mechelen, W., De Vries, N. K., & Brug, J. (2006). Environmental influences on energy balance-related behaviors: a dual-process view. *International Journal of Behavioral Nutrition and Physical Activity, 3*(1), 9.

Lequerica, A. H., & Kortte, K. (2010). Therapeutic engagement: a proposed model of engagement in medical rehabilitation. *American journal of physical medicine & rehabilitation, 89*(5), 415-422.

Leventhal, H., Brissette, I., & Leventhal, E. A. (2003). The common-sense model of self-regulation of health and illness. *The self-regulation of health and illness behaviour, 1*, 42-65.

Lewin, K. (1951). *Field theory in social science: Selected theoretical papers*. New York: Harper & Row.

Locke, E. A., & Latham, G. P. (2002). Building a Practically Useful Theory of Goal Setting and Task Motivation. A 35 Year Odyssey. *American Psychologist, 57*(9), 705-717.

Markus, H. (1977). Self-schemata and processing information about self. *Journal of Personality and Social Psychology, 35*(2), 63-78. doi:10.1037//0022-3514.35.2.63

Marlatt, G. A., & Donovan, D. M. (2005). *Relapse prevention: Maintenance strategies in the treatment of addictive behaviors*: Guilford Press.

Marlatt, G. A., & George, W. H. (1984). Relapse prevention - introduction and overview of the model. *British Journal of Addiction, 79*(3), 261-273. Retrieved from <Go to ISI>://WOS:A1984TK29700004

May, C., & Finch, T. (2009). Implementing, Embedding, and Integrating Practices: An Outline of Normalization Process Theory. *Sociology-the Journal of the British Sociological Association, 43*(3), 535-554. doi:10.1177/0038038509103208

May, C. R., Mair, F., Finch, T., MacFarlane, A., Dowrick, C., Treweek, S., . . . Montori, V. M. (2009). Development of a theory of implementation and integration: Normalization Process Theory. *Implementation Science, 4*. doi:10.1186/1748-5908-4-29

McGuire, W. J. (1976). Some internal psychological factors influencing consumer choice. *Journal of Consumer Research*, 302-319.

McKinlay, J. B. (1995). The new public health approach to improving physical activity and autonomy in older populations *Preparation for aging* (pp. 87-103): Springer.

McLeroy, K. R., Bibeau, D., Steckler, A., & Glanz, K. (1988). An ecological perspective on health promotion programs. *Health Education Behavior, 15*(4), 351-377.

Michie, S., Campbell, R., Brown, J., & West, R. (2014). *ABC of Behaviour Change Theories*. London: Silverback Publishing.

Michie, S., van Stralen, M. M., & West, R. (2011). The behaviour change wheel: a new method for characterising and designing behaviour change interventions. *Implementation Science, 6*(1), 42.

Moss, A. C., & Albery, I. P. (2009). A Dual-Process Model of the Alcohol-Behavior Link for Social Drinking. *Psychological Bulletin, 135*(4), 516-530. doi:10.1037/a0015991

Muraven, M., & Baumeister, R. F. (2000). Self-Regulation and Depletion of Limited Resources: Does Self-Control Resemble a Muscle? *Psychological Bulletin, 126*(2), 247-259. doi:I0.I037//0033-2909.126.2.247

Neff, J. A., & MacMaster, S. A. (2005). Applying behaviour change models to understand spiritual mechanisms underlying change in substance abuse treatment. *The American Journal of Drug and Alcohol Abuse, 31*, 669-684.

Nigg, C. R., Borrelli, B., Maddock, J., & Dishman, R. K. (2008). A theory of physical activity maintenance. *Applied psychology, 57*(4), 544-560.

Odutolu, O. (2005). Convergence of behaviour change models for AIDS risk reduction in sub-Saharan Africa. *The International journal of health planning and management, 20*(3), 239-252.

Ogden, J., & Hills, L. (2008). Understanding sustained behavior change: the role of life crises and the process of reinvention. *Health, 12*(4), 419-437. doi:10.1177/1363459308094417

Ouellette, J. A., & Wood, W. (1998). Habit and intention in everyday life: the multiple processes by which past behavior predicts future behavior. *Psychological Bulletin, 124*(1), 54.

Panter-Brick, C., Clarke, S. E., Lomas, H., Pinder, M., & Lindsay, S. W. (2006). Culturally compelling strategies for behaviour change: A social ecology model and case study in malaria prevention. *Social Science & Medicine, 62,* 2810-2825.

Papa, M. J., Singhal, A., & Papa, W. H. (2006). *Organizing for social change: A dialectic journey of theory and praxis*: Sage.

Petty, R. E., & Cacioppo, J. T. (1986). The elaboration likelihood model of persuasion. *Advances in experimental social psychology, 19*, 123-205.

Polivy, J., & Herman, C. P. (1985). Dieting and binge eating: A causal analysis. . *American Psychologist, 40*, 193–204.

Prochaska, J. O., & Di Clemente, C. C. (1983). Stages and processes of self-change of smoking: Toward an integrative model of change. *Journal of Consulting and Clinical Psychology & Health, 51*(3), 390-395.

Prochaska, J. O., DiClemente, C., C. , & Norcross, J. C. (1992). In search of how people change: Applications to addictive behaviors. *American Psychologist, 47*(9), 1102-1114.

Rogers, E. M. (2010). *Diffusion of innovations*: Simon and Schuster.

Rogers, R. W. (1975). A protection motivation theory of fear appeals and attitude change1. *The journal of psychology, 91*(1), 93-114.

Rogers, R. W., & Prentice-Dunn, S. (1997). Protection motivation theory.

Rokeach, M. (1968). Beliefs, attitudes and values: A theory of organization and change.

Romer, D., & Hornik, R. (1992). HIV education for youth: the importance of social consensus in behaviour change. *AIDS care, 4*(3), 285-303.

Rosenstock, I. M. (1974). Historical origins of the health belief model. *Health Education & Behavior, 2*(4), 328-335.

Rothman, A. J. (2000). Toward a theory-based analysis of behavioral maintenance. *Health Psychology, 19*(1), 64-69. doi:10.1037//0278-6133.19.Suppl1.64

Rothman, A. J., Baldwin, A. S., & Hertel, A. W. (2004). Self-regulation and Behavior Change: Disentangling Behavioral Initiation and Behavioral Maintenance In R. F. Baumeister & K. D. Vohs (Eds.), *Handbook of Self-Regulation* (pp. 130-1150). London: The Gulford Press.

Rothman, A. J., Sheeran, P., & Wood, W. (2009). Reflective and Automatic Processes in the Initiation and Maintenance of Dietary Change. *Annals of Behavioral Medicine, 38,* S4-S17. doi:10.1007/s12160-009-9118-3

Ryan, R. M., & Deci, E. L. (2000). Self-determination theory and the facilitation of intrinsic motivation, social development and well-being. *American Psychologist, 55*, 68-78.

Schulz, A., & Northridge, M. E. (2004). Social determinants of health: implications for environmental health promotion. *Health Education & Behavior, 31*(4), 455-471.

Schwarzer, R. (1992). Self-efficacy in the adoption and maintenance of health behaviors: Theoretical approaches and a new model. In R. Schwarzer (Ed.), *Selfefficacy: Thought control of action* (pp. 217-243). Washington, DC: Hemisphere.

Schwarzer, R. (2008). Modeling health behavior change: How to predict and modify the adoption and maintenance of health behaviors. *Applied Psychology-an International Review-Psychologie Appliquee-Revue Internationale, 57*(1), 1-29. doi:10.1111/j.1464-0597.2007.00325.x

Skinner, B. F. (1953). *Science and human behaviour.* New York: Macmillan.

Sniehotta, F. F., Schwarzer, R., Scholz, U., & Schuz, B. (2005). Action planning and coping planning for long-term lifestyle change: Theory and assessment. *European Journal of Social Psychology, 35*(4), 565-576. doi:10.1002/ejsp.258

Stasiewicz, P. R., & Maisto, S. A. (1993). Two-Factor Avoidance Theory: The Role of Negative Affect in the Maintenance of Substance Use and Substance Use Disorder. *Behavior Therapy, 24*, 337-356.

Stevens, M., Bult, P., de Greef, M. H. G., Lemmink, K., & Rispens, P. (1999). Groningen active living model (GALM): Stimulating physical activity in sedentary older adults. *Preventive Medicine, 29*(4), 267-276. doi:10.1006/pmed.1999.0545

Stokols, D. (1992). Establishing and maintaining healthy environments: toward a social ecology of health promotion. *American Psychologist, 47*(1), 6.

Strack, F., & Deutsch, R. (2004). Reflective and impulsive determinants of social behavior. *Personality and Social Psychology Review, 8*(3), 220-247.

Stroebe, W., Mensink, W., Aarts, H., Schut, H., & Kruglanski, A. W. (2008). Why dieters fail: Testing the goal conflict model of eating. *Journal of Experimental Social Psychology, 44*(1), 26-36. doi:10.1016/j.jesp.2007.01.005

Swinburn, B., Egger, G., & Raza, F. (1999). Dissecting obesogenic environments: the development and application of a framework for identifying and prioritizing environmental interventions for obesity. *Preventive Medicine, 29*(6), 563-570.

Tajfel, H., & Turner, J. (1979). An integrative theory of intergroup conflict. In W. G. Austin & S. Worchel (Eds.), *The Social Psychology of Intergroup Relations*. Monterey, CA: Brooks-Cole.

Thompson, B., & Kinne, S. (1990). Social change theory: applications to community health. In N. Bracht (Ed.), *Health promotion at the community level*. Newbury Park: Sage Publications.

Triandis, H. C. (1977). *Interpersonal behavior*. Monterey: Brooks/Cole Publishing Company.

Vancouver, J. B., & Kendall, L. N. (2006). When self-efficacy negatively relates to motivation and performance in a learning context. *Journal of Applied Psychology, 91*(5), 1146.

Vancouver, J. B., Thompson, C. M., Tischner, E. C., & Putka, D. J. (2002). Two studies examining the negative effect of self-efficacy on performance. *Journal of Applied Psychology, 87*(3), 506.

Vaughan, P. W., & Rogers, E. M. (2000). A staged model of communication effects: Evidence from an entertainment-education radio soap opera in Tanzania. *Journal of health communication, 5*(3), 203-227.

Venkatesh, V., & Bala, H. (2008). Technology acceptance model 3 and a research agenda on interventions. *Decision sciences, 39*(2), 273-315.

Venkatesh, V., & Davis, F. D. (2000). A theoretical extension of the technology acceptance model: four longitudinal field studies. *Management science, 46*(2), 186-204.

Venkatesh, V., Morris, M. G., Davis, G. B., & Davis, F. D. (2003). User acceptance of information technology: Toward a unified view. *MIS quarterly*, 425-478.

Verplanken, B. (2006). Beyond frequency: Habit as mental construct. *British Journal of Social Psychology, 45*, 639-656. doi:10.1348/014466605x49122

Verplanken, B., & Aarts, H. (1999). Habit, Attitude, and Planned Behaviour: Is Habit an Empty Construct or an Interesting Case of Goal-directed Automaticity? *European Review of Social Psychology, 10*(1), 101-134. doi:10.1080/14792779943000035

Verplanken, B., & Orbell, S. (2003). Reflections on past behavior: A self-report index of habit strength. *Journal of Applied Social Psychology, 33*(6), 1313-1330. doi:10.1111/j.1559-1816.2003.tb01951.x

Verplanken, B., Walker, I., Davis, A., & Jurasek, M. (2008). Context change and travel mode choice: Combining the habit discontinuity and self-activation hypotheses. *Journal of Environmental Psychology, 28*(2), 121-127. doi:10.1016/j.jenvp.2007.10.005

Wallerstein, N., & Sanchez-Merki, V. (1994). Freirian praxis in health education: research results from an adolescent prevention program. *Health Education Research, 9*(1), 105-118.

Weinstein, N. D. (1988). The Precaution Adoption Process. *Health Psychology, 7*(4), 355-386. doi:10.1037//0278-6133.7.4.355

Weinstein, N. D., & Sandman, P. M. (1992). A model of the precaution adoption process - evidence from home radon testing *Health Psychology, 11*(3), 170-180. doi:10.1037/0278-6133.11.3.170

West, R., & Brown, J. (2013). *Theory of addiction*: John Wiley & Sons.

Westaby, J. D. (2005). Behavioral reasoning theory: Identifying new linkages underlying intentions and behavior. *Organizational Behavior and Human Decision Processes, 98*(2), 97-120.

Wight, D., Abraham, C., & Scott, S. (1998). Towards a psycho-social theoretical framework for sexual health promotion. *Health Education Research, 13*(3), 317-330.

Witte, K. (1992). Putting the fear back into fear appeals: The extended parallel process model. *Communications Monographs*, *59*(4), 329-349.

Appendix 3. Theories assessed in the review (On-line supplement)

ID	Theory name and references used	Theory intended application (according to theory authors)	Allocated theme	Behaviour specific (yes/no) if yes – which behaviour	Population specific (yes/no) if yes which population	Times suggested
1	Active living model (Stevens, Bult, de Greef, Lemmink, & Rispens, 1999)	A behavioural change strategy for stimulating physical activity participation	**Maintenance motives** (enjoyment of behaviour)	Yes Leisure-time physical activity	Yes Sedentary older adults	0
2	Affect infusion model (Forgas, 1995, 2001)*	A comprehensive account of the role of affective states in social judgments	None	No	No	0
3	AIDS risk reduction model (Catania, Kegeles, & Coates, 1990)	A model of AIDS Risk Reduction Process	[Learning process]	Yes Sexual behaviour; Learning	Yes Adults at risk of being infected with HIV	0
4	Attitude-social influence-efficacy model/integrated change model (De Vries et al., 2000; De Vries, Mesters, Steeg, & Honing, 2005)	A model of motivational change	[Motives]	Yes Health-related behaviour	No	2
5	Attribution theory (Jones, 1976)*	A theory of the process by which people form causal interpretations of the events around them	None	No	No	2

6	Behavioural reasoning theory (Westaby, 2005)	Extension of TPB, explains conscious behaviour	[Motives]	No	No	0
7	Belief systems theory (Rokeach, 1968)	A theory of organization and change within value-attitude systems	[Motives]	No	No	0
8	Classical conditioning (Skinner, 1953)	A learning theory; explains animal and human behaviour	[Learning process]	Yes Learning	No	4
9	COM-B system (Susan Michie, van Stralen, & West, 2011)	A model of behaviour, also provides a basis for designing interventions aimed at behaviour change	[Motives; Resources and Environment]	No	No	0
10	Common sense self-regulation model (Leventhal, Brissette, & Leventhal, 2003)	A model of illness representations	**Self-regulation**	No	No	3
11	Control theory (Carver & Scheier, 1982)	A theory explains how people perceive the environment they live in and how they react to the environmental changes	**Self-regulation**	No	No	2
12	Convergence of behaviour change models for AIDS risk reduction in Sub-Saharan Africa (Odutolu, 2005)	A comprehensive model of AIDS risk reduction theories in sub-Saharan Africa	[Motives, Environment, social influence and social change]	Yes Sexual behaviour	Yes People in sub-Saharan Africa	0
13	Demand-control model (job strain model) (Karasek, 1979)*	A model of the impact of the work environment on life outside the job	None	Yes behaviour performed within work environment	Yes People who work	0

14	Developmental causal model of the process of becoming a smoker (Flay, d'Avernas, Best, Kersell, & Ryan, 1983)	A model of a process of becoming a smoker	**Habit** [Learning process]	Yes Smoking	Yes Young people	0
15	Diffusion of innovations theory (Rogers, 2010)	A theory of diffusion of innovations	**Self-regulation** [Learning process]	Yes Adapting innovations	No	2
16	Dual process model of alcohol-behaviour link (Moss & Albery, 2009)	A dual process model of the alcohol-behaviour link for social drinking.	[Environment, social influence and social change]	Yes Drinking alcohol	No	0
17	Ecological model of health behaviours (McLeroy, Bibeau, Steckler, & Glanz, 1988)	A model provides an ecological perspective on health promotion programs	[Environment, social influence and social change]	Yes Health-related behaviour	No	0
18	Ecological systems theory (Bronfenbrenner, 1977, 1986)	An ecological theory: person is embedded in the multilevel system which influences behaviour	[Environment, social influence and social change]	No	No	1
19	Elaboration likelihood model (Petty & Cacioppo, 1986)*	A general theory of persuasion	None	No	No	0
20	Extended information processing model (Flay, DiTecco, & Schlegel, 1980)*	A model of the extended human information processing including attention and memory.	None	No	No	0
21	Extended parallel process model (Witte, 1992)*	A theory of fear appeal	None	No	No	0
22	Family systems theory (Bowen, 1966)	A theory of family systems	[Environment, social influence	No	No	0

			and social change]			
23	Goal theory (Bagozzi, 1992)	A theory explains the processes that occur between intentions and goal-directed behaviours	[Self-regulation]	No	No	0
24	Goal setting theory (Locke & Latham, 2002)	A theory based on Ryan's (1970) premise that conscious goals affect action	**Self-regulation**	No	No	2
25	Health Action Process Approach (Schwarzer, 1992, 2008; Sniehotta, Schwarzer, Scholz, & Schuz, 2005)	A psychological theory of health behavior change	**Self-regulation**	Yes Health-related behaviour	No	**10**
26	Health behaviour goal model (Gebhardt, 1997)	A model is an attempt to describe and predict the process of behaviour change	[Motives]	Yes Health-related behaviour	No	0
27	Health belief model (Rosenstock, 1974)	A model of individual actions which should be taken to avoid a disease	[Motives]	Yes Health-related behaviour	No	3
28	Health capital theory (Grossman, 1972)*	Theory of health capital: health is a durable capital stock that produces an output of healthy time	None	Yes Health-related behaviour	No	0
29	Health-related model of behaviour change (Hunt & Martin, 1988)	A model of activities which are habitually performed and become routine	**Habit**	Yes Health-related behaviour	No	0
30	Information-motivation-behavioural skills model	A model which is a conceptualization of AIDS-risk behaviour change	[Motives]	Yes Sexual behaviour	No	1

	(Fisher, Fisher, Williams, & Malloy, 1994)					
31	Integrated theoretical model for alcohol and drug prevention (Gonzalez, 1989)	A theory of alcohol and other drug abuse prevention for higher education	[Motives]	Yes<br>Alcohol and drug use	Yes<br>Students	0
32	Integration of Freire and protection motivation theory (Wallerstein & Sanchez-Merki, 1994)	A comprehensive adolescent social action program	[Motives]	Yes<br>Learning	Yes<br>People in educational settings	0
33	Integrative conceptual model of spiritual mechanisms underlying substance abuse behaviour change (Neff & MacMaster, 2005)	A framework for viewing substance abuse treatment and change in substance abuse behaviours during treatment	Environment, **social influence** and social change	Yes<br>Substance abuse	Yes<br>People who believe in God	0
34	Integrative model (Fishbein, 2000)	An integration of theories of behavioural prediction and behaviour change	[Motives]	Yes<br>Sexual behaviour	No	0
35	Integrative model of health attitude and behaviour change (Flay et al., 1983)	A model of the communication process	[Learning process]	Yes<br>Smoking	Yes<br>Young people	0
36	Model of human occupation (Kielhofner, 2002)	A model of human volition, habituation and performance capacity	**Habit**	No	No	0
37	Multi-level/multi-media model of social change (Dresler-Hawke & Veer, 2006)	A model of behaviour, environmental change and public policy for healthy choices	[Environment, social influence and social change]	No	No	0

38	Needs-opportunities-abilities model (Gatersleben & Vlek, 1998)	A model of consumer behaviour	[Motives]	Yes<br>Consumer behaviour	Yes<br>Consumers	0
39	Net-present value economic theory (Wight, Abraham, & Scott, 1998)*	A theory used in economics to analyse potential investment	None	Yes<br>Investment	No	0
40	Network theory of collective action (Heckathorn, 1990)	Theory of group-mediated social control	[Environment, social influence and social change]	No	No	0
41	Operant learning theory (Skinner, 1953)	Leading learning theory; explains reflective learning	[Learning process]	No	No	2
42	Precaution adoption process model (Weinstein, 1988; Weinstein & Sandman, 1992)	A stage theory of preventative behaviour	[Motives]	Yes<br>Preventive behaviour	No	2
43	Pressure system model (Katz, 2001)	A stage model for sequential assessments of the balance between resistance and motivation	**Self-regulation**	Yes<br>Primary care	Yes<br>Patients	0
44	PRIME theory of motivation (West & Brown, 2013)	The theory of motivation, including: plans, responses, impulses, motives and evaluations	[Motives]	No	No	0
45	Problem behaviour theory (Jessor & Jessor, 1977)	The theory of relationships between personality, perceived environment and behaviour	[Motives; Environment]	Yes<br>Risk behaviours	Yes<br>Young people	0

46	Protection motivation theory (Rogers, 1975; Rogers & Prentice-Dunn, 1997)	A theory of fear appeal	[Motives]	No	No	3
47	Prototype willingness model (Gerrard, Gibbons, Houlihan, Stock, & Pomery, 2008)	A model of decision making involved in health behavior	[Motives]	Yes<br>Risk behaviours	Yes<br>Young people	1
48	Reflective and Impulsive Model (Strack & Deutsch, 2004)	A dual process model including a reflective system and an impulsive system	**Self-regulation**<br>**Habit**<br>**Resources**	No	No	0
49	Regulatory fit theory (Higgins, 2006)	A theory of regulatory fit ('Fit makes people engage more strongly in what they are doing and feel right about it.')	**Maintenance motives** (satisfaction with behaviour)	No	No	0
50	Salutogenic model (Antonovsky, 1996)*	A model of a salutogenic orientation as the basis for health promotion	None	Yes<br>Health promotion	No	0
51	Self-determination theory (Deci & Ryan, 2002; Deci & Ryan, 2010; Ryan & Deci, 2000)	A theory of the processes through which a person acquires the motivation for initiating new health-related behaviours and maintaining them over time	**Maintenance motives** (satisfaction with outcomes)	Yes<br>Health-related behaviours	No	**7**
52	Self-efficacy theory (Bandura, 1977)	A theory of perceived self-efficacy	[Motives]	No	No	0
53	Self-perception theory (Bem, 1967, 1972)	A theory of self-attitudes	**Maintenance motives** (identity)	No	No	0

54	Self-regulation theory (Kanfer & Gaelick-Buys, 1991)	A theory of the initial stages of behaviour change, focusing on the detection of maladaptive behaviour and the early sources of motivation for change	**Self-regulation**	No	No	0
55	Six staged model of communication effects (Vaughan & Rogers, 2000)	A model of the adoption of family planning methods	**Maintenance motives** (satisfaction with outcomes)	Yes Family planning	Yes Young people	0
56	Social action theory (Ewart, 1991)	An integrative action schema for defining public health goals and identifying changeable influences to encourage self-protective activities	[Environment, social influence and social change]	Yes Health-related behaviours	No	0
57	Social change theory (Thompson & Kinne, 1990)	A theory for changing community norms about health related behaviour	Environment, social influence and **social change**	No	No	0
58	Social change theory of dialogue and praxis (Papa, Singhal, & Papa, 2006)*	A theory of community organisation and social change explaining dialogue and praxis	None	No	No	0
59	Social cognitive/learning theory (Bandura, 1986)	An interactional model of causation in which environmental events, personal factors, and	Environment, **social influence** and social change	No	No	**9**

		behavior all operate as interacting determinants of each other				
60	Social consensus model of health education (Romer & Hornik, 1992)	A model of social consensus in behaviour change for HIV prevention	[Environment, social influence and social change]	Yes<br>Sexual behaviours	Yes<br>Young people	0
61	Social development model (Hawkins & Weis, 1985)	A model of delinquency prevention derived from integrating control<br>and social learning theories	[Environment, social influence and social change]	Yes<br>'Delinquent behaviour'	Yes<br>Young people	0
62	Social ecological model of health promotion (Stokols, 1992)	A model of the development of effective strategies to promote personal<br>and collective well-being	[Environment, social influence and social change]	Yes<br>Health promotion	No	0
63	Social ecological model of walking (Alfonzo, 2005)	A model for the decision-making process of walking	[Environment, social influence and social change]	Yes<br>Walking	No	0
64	Social ecology model of behaviour change (Panter-Brick, Clarke, Lomas, Pinder, & Lindsay, 2006)	A social ecology perspective to make explicit the links between intention to change, actual behaviour change, and subsequent health impact	[Environment, social influence and social change]	No	No	0
65	Social identity model (Tajfel & Turner, 1979)	The theory defines 'social identity'	[Environment, social influence and social change]	No	No	1

66	Social norms theory (Berkowitz, 2004)	A theory of social norms which has implications for health promotion and prevention	[Motives; Environment, social support and social change]	Yes Health promotion and prevention	No	0
67	Technology acceptance model 1 (Davis, 1989)	A model of technology acceptance	[Motives]	Yes Technology acceptance	No	0
68	Technology acceptance model 2 (Venkatesh & Davis, 2000)	A model of technology acceptance	[Motives]	Yes Technology acceptance	No	0
69	Technology acceptance model 3 (Venkatesh & Bala, 2008)	A model of technology acceptance	**Maintenance motives** (satisfaction with outcomes)	Yes Technology acceptance	No	0
70	Temporal self-regulation model (Hall & Fong, 2007)	A theory focusing on the capacity to engage in behaviour	**Maintenance motives** (satisfaction with outcomes)	No	No	0
71	Theoretical framework for behaviour change (Burnet, Plaut, Courtney, & Chin, 2002)	A framework for environmental influences on behaviour as well as intrapersonal determinants	[Motives; Environment, social support and social change]	Yes Diabetes prevention	Yes Young people	0
72	Theories of reasoned action/theory of planned behaviour (Ajzen, 1985; Fishbein & Ajzen, 1975)	A theory designed to predict volitional behaviours and to understand their psychological determinants	[Motives; Environment, social support	No	No	4

			and social change]			
73	Theory of change (Lewin, 1951)	A theory which describes change as ‘an on-going social process’	[Environment, social support and social change]	No	No	0
74	Theory of interpersonal behaviour (Triandis, 1977)	A theory of interpersonal behaviour	**Self-regulation Habit**	No	No	0
75	Theory of rational addiction (Becker & Murphy, 1988)*	A theory of rational addiction: addictions (to heroin, tobacco, television, etc.) can be usefully modelled as specific kinds of rational, forward-looking, optimal consumption plans	None	Yes Addictive behaviours	No	0
76	Transcontextual model of motivation (Hagger, Chatzisarantis, Culverhouse, & Biddle, 2003)	a theory of social cognition to explain the psychological processes by which young people transfer motivation during physical education into physical activity behaviour during leisure time	[Motives]	Yes Exercise	Yes Young people	0
77	Transtheoretical/stages of change model (Prochaska & Di Clemente, 1983; Prochaska, DiClemente, & Norcross, 1992)	A theory which originally emerged from a comparative analysis of 18 leading therapy systems	Environment, **social influence** and social change	Yes Smoking	No	**11**
78	Unified theory of acceptance and use of technology	A theory of acceptance and use of technology	[Motives]	Yes	No	0

	(Venkatesh, Morris, Davis, & Davis, 2003)			Acceptance and Use of Technology		
79	Utility theory (Fishburn, 1968)*	A theory of people's preferences or values and assumptions about a person's preferences that enable them to be represented in numerically useful ways	None	No	No	0
80	Yale information processing model (McGuire, 1976)*	A model of the variables that might influence comprehension, acceptance, and retention of persuasive messages	None	No	No	0
81	Model of behaviour maintenance (Rothman, 2000; Rothman, Baldwin, & Hertel, 2004)	A theory of behaviour change maintenance	**Maintenance motives** (satisfaction with outcomes)	No	No	**8**
82	Relapse prevention theory (Marlatt & Donovan, 2005; Marlatt & George, 1984)	A theory of preventing relapse and coping with situational barriers	**Self-regulation**	Yes Health behaviour (preventing relapse)	No	**9**
83	Strength model of self-control (Baumeister, Bratslavsky, Muraven, & Tice, 1998; Baumeister, Muraven, & Tice,	A theory of self-regulation as a limited cognitive resource	**Resources**	No	No	0

	2000; Muraven & Baumeister, 2000)					
84	Goal conflict model (Stroebe, Mensink, Aarts, Schut, & Kruglanski, 2008)	A theory of eating regulation	**Resources**	Yes<br>Eating (dieting)	No	1
85	Habit theory (Verplanken, 2006; Verplanken & Aarts, 1999; Verplanken & Orbell, 2003; Verplanken, Walker, Davis, & Jurasek, 2008)	A theory of habit; habit is defined as a psychological construct, rather than simply past behavioural frequency; refers to 'habitual mind-sets'	**Habit**	Yes<br>Habitual behaviours	No	**6**
86	Theory of physical activity maintenance (Nigg, Borrelli, Maddock, & Dishman, 2008)	A theory of physical activity maintenance	**Maintenance motives**<br>**Self-regulation**	Yes<br>Physical activity	No	3
87	Rubicon model of action phases (Heckhausen, 2007)	A stage model of behaviour which is divided into two phases: a motivation phase and a volition phase	**Self-regulation**	No	No	2
88	Intrinsic motivation and cognitive evaluation theory (Hoss, 1985)*	A theory of self-motivation and underlying cognitive processes	None	No	No	0
89	Two-factor avoidance theory (Stasiewicz & Maisto, 1993)	A learning-based explanation of the role of negative affect in the maintenance of substance use disorder	[Learning process]	Yes<br>Substance use	No	0
90	The dietary restraint theory (Polivy & Herman, 1985)	A theory of eating regulation	**Resources**	Yes<br>Eating (dieting)	No	0

91	Process model of lifestyle behaviour change (Greaves, Reddy, & Sheppard, 2010)	A process model for supporting a lifestyle behaviour change	**Self-regulation Habit**	Yes Health-related behaviour	No	1
92	Self-schema theory (Markus, 1977)	A theory of 'self-schema' - the beliefs and ideas people have about themselves	**Maintenance motives** (identity)	No	No	1
93	Habit and intention theory (Ouellette & Wood, 1998)	A theory of habit	**Habit**	Yes Health-related behaviours	No	2
94	Selection, optimisation and compensation model (Baltes, 1997)	A model of successful development and aging	**Resources**	Yes Aging	Yes Older people	1
95	Normalisation process theory (May & Finch, 2009; May et al., 2009)	A set of sociological tools to explain how new or modified practices are operationalized in healthcare and other institutional settings	**Environment**, social influence and **social change**	Yes Health care	Yes Population within health-care and other institutional settings	1
96	RE-AIM framework (Glasgow, Vogt, & Boles, 1999)*	A sociological framework for designing interventions	None	Yes Health-related behaviours	No	1
97	Dual process model of self-control (Friese, Hofmann, & Wanke, 2008; Hofmann, Friese, & Wiers, 2008)	A model of impulsive and reflective influences on health behaviour	**Resources Self-regulation Habits**	Yes Health-related behaviours	No	2
98	Model of engagement in medical rehabilitation (Lequerica & Kortte, 2010)	A model of patient engagement in rehabilitation	**Self-regulation**	Yes Rehabilitation	Yes Patients	1

99	Three-factor model to guide practice (DiMatteo, Haskard-Zolnierek, & Martin, 2012)	A brief narrative review of research on (non)adherence and three-factor heuristic model to guide practitioners	**Self-regulation** Social influence	Yes Medical non-adherence	Yes Patients	1
100	A 2 x 2 behaviour change matrix (Rothman, Sheeran, & Wood, 2009)	A theory decomposing action control and behavior change into a 2 (reflective, automatic) ×2 (initiation, maintenance) matrix	**Maintenance motives** (satisfaction with outcomes)	Yes Food choice	No	2
101	Coping planning (Sniehotta et al., 2005)	A theory of the mental simulation of overcoming anticipated barriers to action.	**Self-regulation**	Yes Health-related behaviour	No	3
102	Pavlovian instrumental transfer (Balleine & Killcross, 2006; Corbit & Balleine, 2005)	A learning theory: in instrumental conditioning, presentation of a reinforcer is contingent upon the performance of a specific action by the subject	[Learning process]	Yes Learning	No	1
103	Associative theories of goal-directed behaviour (de Wit & Dickinson, 2009)	A theory of human decision-making	[Learning process]	Yes Learning	No	1
104	Population-based health promotion model (McKinlay, 1995)	An environmental model focusing on PA in sedentary older adults	[Motives; Environment, social support and social change]	Yes Physical activity	Yes Sedentary older adults	1
105	The operation of the basal ganglia (Graybiel, 1995, 2005)	A learning neuronal model: basal ganglia are critically involved in building up sequences of	[Learning process]	Yes Learning	No	1

		behavior into meaningful, goal-directed action				
106	Revision and persistence (Vancouver & Kendall, 2006; Vancouver, Thompson, Tischner, & Putka, 2002)	A theory of behavioural goals defined as internal representations of desired states	[Motives]	No	No	1
107	Organizational change theory (Cummings & Worley, 2014)	A theory of organization development intervention	[Environment, social support and social change]	Yes<br>Organizational change	Yes<br>People within the organisation	1
108	Self-regulatory theories (Boekaerts, 1997)	A theory of self-regulated learning	[Self-regulation]	Yes<br>Learning	No	1
109	A behavioral model of medication adherence (De Bruin, Hosters, Van Den Borne, Kok, & Prins, 2005)	A behavioural model of medication adherence	**Self-regulation**<br>**Maintenance motives** (satisfaction with outcomes)	Yes<br>adherence	Yes<br>Patients	2
110	Self-concept theory (Bracken, 1996)	A theory of self-perception	**Maintenance motives** (identity)	No	No	1
111	A learning theory perspective on lapse, relapse, and the maintenance of behavior change (Bouton, 2000)	A learning theory perspective on lapse, relapse, and the maintenance of behavior change	**Self-regulation** [Learning process]	Yes<br>Learning	No	1
112	Health behavior internalization model (Bellg, 2003)	A model of motivational factors associated with internalization processes	**Maintenance motives** (identity)	Yes<br>Health-related behaviour	No	2

113	Angelo (Swinburn, Egger, & Raza, 1999)*	An environmental framework focusing on behavior sustainability factors: analysis grid for environments linked to obesity	None	Yes<br>Eating	Yes<br>Obese people	1
114	Social determinants of health and environmental health promotion model (Schulz & Northridge, 2004)	A conceptual framework for environmental health promotion	[Environment, social support and social change]	Yes<br>Health-related behaviour	No	1
115	Environmental research framework for weight gain prevention (Kremers et al., 2006)*	An environmental research framework for weight gain prevention; A dual-process model	None	Yes<br>Eating	No	1
116	Life crises and the process of reinvention theory (Epiphaniou & Ogden, 2010; Ogden & Hills, 2008)	A theory of sustained behavior change triggered by a significant life crisis relating to health, relationships or salient milestones	**Maintenance motives** (life changing events)	Yes<br>Health-related behaviour	No	2
117	The strength model of self-control (Hagger, Wood, Stiff, & Chatzisarantis, 2009)	The strength model of self-regulation failure and health-related behaviour	**Resources** and **self-regulation**	Yes<br>Health-related behaviour	No	1

Themes which are written in the squared brackets refer to the theories which fit perfectly into our theory review themes but give equal explanations/predictions for behaviour initiation and behaviour change maintenance. Times suggested: How many times suggested by theory experts. Theories marked with star were not included in data synthesis (reasons explained in text).

Appendix 4. Theory theme validation exercise (On-line supplement)

Instructions: Thank you very much for agreeing to take part in this theme validation exercise. This task should take about 15 minutes. Please read the theme definitions carefully.

Short theme definitions:

A. **Maintenance motives** – reasons why people engage in behaviour.
B. **Resources** – psychological and physical assets that someone can draw on in order to engage in behaviour.
C. **Self-regulation** – ability to actively control behaviour; ability to override or inhibit behaviours, urges, emotions or desires that would otherwise hinder goal-directed behaviour.
D. **Habits** - behavioural patterns, based on context-behaviour associations which are learned through context-dependent repetition. They are elicited automatically when associated contexts are encountered.
E. **Environment and social influences**- the setting or conditions in which a particular activity is carried on; these may include social settings, social support and social change.

Please allocate each extracted theory statement to the theme it fits in (in your opinion). You can allocate one statement to more than one theme.

Statements examples:

1. 'The social reinforcements obtained from smoking are probably the most important influence on whether or not an experimenting adolescent will become a regular smoker. Peer pressure still may play a role as this stage, although its effects probably are mediated through social reinforcement.'

2. ‘We consider various mechanisms that could underlie the habitual control of action, and we conclude that direct cuing and motivated contexts best account for the characteristic features of habit responding—in particular, for the rigid repetition of action that can be initiated without intention and that runs to completion with minimal conscious control’

3. ‘The term self-schema refers to the beliefs and ideas people have about themselves. These beliefs are used to guide and organize information processing, especially when the information is significant to the self. Self-schemas are important to a person's overall self-concept. The term schematic describes having a particular schema for a particular dimension. For instance, a person in a rock band at night would have a "rocker" schema. However, during the day, if he works as a salesperson, he would have a "salesperson" schema during that period of time.’

4. ‘The authors review evidence that self-control may consume a limited resource. Exerting self-control may consume self-control strength, reducing the amount of strength available for subsequent self-control efforts. Coping with stress, regulating negative affect, and resisting temptations require self-control, and after such self-control efforts, subsequent attempts at self-control are more likely to fail. Continuous self-control efforts, such as vigilance, also degrade over time.’

5. ‘[…] relapse is not viewed merely as an indicator of treatment failure. Instead, potential and actual episodes are key targets for both proactive and reactive intervention strategies. RP treatment procedures include specific intervention techniques designed to teach the individual to effectively anticipate and cope with potential relapse situations.’

6. ‘Decisions regarding behavioral initiation are predicted to depend on favorable

expectations regarding future outcomes, whereas decisions regarding behavioral maintenance are predicted to depend on perceived satisfaction with received outcomes.'

7. 'In our model we also assume that a warm, trusting relationship is a precondition not only for therapy but for any of the helping professions. If clients feel that the professional does not care about them or if they do not trust the professional to adequately care for their needs, then clients are obviously more likely to terminate the helping relationship rather quickly'

8. 'Just as preparation for action was essential for success, so too was preparation for maintenance. Successful maintenance builds on each of the processes that came before. Specific preparation for maintenance entailed an assessment of the conditions under which a person was likely to relapse and development of alternative responses for coping with such conditions without resorting to self-defeating defences and pathological responses.'

9. 'There also appears to be general agreement among therapists, but less conclusive support, that motivation for change is a key precondition for therapy.'

10. '[…] under conditions of low control resources, automatic affective reactions toward tempting stimuli should exert a stronger influence on health behavior than under conditions of full resource availability. In a complementary manner, the impact of reasoned attitudes or restraint standards should be stronger under full resources and wane with increasing processing strain on reflective operations.'

11. 'Whereas behavioral initiation is thought to be based on expectations about future outcomes, the decision to maintain an adopted pattern of behavior is thought to be based on people's satisfaction with the outcomes they have obtained. The guiding premise is

that people will maintain a change in behavior only if they are satisfied with what they have accomplished.'

12. 'I proposed that people experience regulatory fit when the manner of their engagement in an activity sustains (rather than disrupts) their current motivational orientation or interests. Fit makes people engage more strongly in what they are doing and feel right about it. Individuals, for example, can pursue the same goal with different orientations and in different ways.'
13. 'Intrinsic motivation involves doing a behaviour because the activity itself is interesting and spontaneously satisfying. When intrinsically motivated, people perform activities because of the positive feelings resulting from the activities themselves. People are interested in what they are doing, and they display curiosity, explore novel stimuli, and work to master optimal challenges.'
14. 'Participants with higher levels of coping planning after discharge were more likely to report higher levels of exercise four months after discharge.'
15. 'Failures to change do not necessarily indicate poor willpower or insufficient understanding of health issues but instead the power of situations to trigger past responses. Habits keep us doing what we have always done, despite our best intentions to act otherwise.'
16. 'The family is a system in that a change in one part of the system is followed by compensatory change in other parts of the system.'
17. 'A habit seems to be accompanied by an enduring cognitive orientation, which we refer to as "habitual mind-set" that makes an individual less attentive to new information and courses of action, and thus contributes to the maintenance of habitual behaviour.'

18. ‘People with high goals produce more because they are dissatisfied with less. The bar for their satisfaction is set at a high level. This is why they are motivated to do more than those with easy goals.’

19. ‘Studies in psychophysiology and cognitive psychology provide evidence that activities which are habitually performed become “routine” in such a way that higher levels of cognitive processing is, for most of the time, unnecessary. Such activities are, therefore, carried out with minimum awareness, coming to the forefront of consciousness only occasionally.’

20. ‘In later stages of the process, as the individual incorporates these cognitive changes and acquires behaviour change skills and behavioural intention to change, peer influence processes become increasingly important in reinforcing behavioural changes.’

Please answer the following questions:

1. Do you have a degree in Health Psychology?
    - ☐ Yes
    - ☐ No
2. Are you…:
    - ☐ A PhD student
    - ☐ Researcher
    - ☐ Academic
    - ☐ Other, please specify:

Thank you very much for taking part!
